# A systems approach to mapping transcriptional networks controlling surfactant homeostasis

**DOI:** 10.1186/1471-2164-11-451

**Published:** 2010-07-26

**Authors:** Yan Xu, Minlu Zhang, Yanhua Wang, Pooja Kadambi, Vrushank Dave, Long J Lu, Jeffrey A Whitsett

**Affiliations:** 1 Division of Pulmonary Biology, Department of Pediatrics, Cincinnati Children's Hospital Medical Center, University of Cincinnati College of Medicine, Cincinnati, OH, USA; 2 Division of Biomedical Informatics, Department of Pediatrics, Cincinnati Children's Hospital Medical Center, University of Cincinnati College of Medicine, Cincinnati, OH, USA; 3 Department of Computer Science, University of Cincinnati College of Medicine, Cincinnati, OH, USA; 4 Department of Biomedical Engineering, University of Cincinnati, Cincinnati, OH, USA

## Abstract

**Background:**

Pulmonary surfactant is required for lung function at birth and throughout life. Lung lipid and surfactant homeostasis requires regulation among multi-tiered processes, coordinating the synthesis of surfactant proteins and lipids, their assembly, trafficking, and storage in type II cells of the lung. The mechanisms regulating these interrelated processes are largely unknown.

**Results:**

We integrated mRNA microarray data with array independent knowledge using Gene Ontology (GO) similarity analysis, promoter motif searching, protein interaction and literature mining to elucidate genetic networks regulating lipid related biological processes in lung. A Transcription factor (TF) - target gene (TG) similarity matrix was generated by integrating data from different analytic methods. A scoring function was built to rank the likely TF-TG pairs. Using this strategy, we identified and verified critical components of a transcriptional network directing lipogenesis, lipid trafficking and surfactant homeostasis in the mouse lung.

**Conclusions:**

Within the transcriptional network, SREBP, CEBPA, FOXA2, ETSF, GATA6 and IRF1 were identified as regulatory hubs displaying high connectivity. SREBP, FOXA2 and CEBPA together form a common core regulatory module that controls surfactant lipid homeostasis. The core module cooperates with other factors to regulate lipid metabolism and transport, cell growth and development, cell death and cell mediated immune response. Coordinated interactions of the TFs influence surfactant homeostasis and regulate lung function at birth.

## Background

Pulmonary surfactant is a lipid-protein complex that is synthesized by type II epithelial cells in the alveoli. Surfactant is stored in intracellular organelles known as lamellar bodies and is secreted into airspace by exocytosis. Surfactant lipids form monolayer and multilayer that line the alveolar surface, reducing surface tension created at the air-liquid interface. Pulmonary surfactant is essential for the proper inflation and function of the lung [1]. Surfactant deficiency is associated with premature birth, lung infection or injury. Mutations in genes critical for surfactant production or function can cause lung atelectasis and respiratory failure [2]. Surfactant homeostasis is maintained by a balance among multi-tiered processes, including the synthesis assembly, trafficking, storage, secretion recycling and degradation of surfactant proteins and lipids. While the structures and functions of pulmonary surfactant proteins and lipids have been extensively studied, little is known regarding the genetic and cellular mechanisms integrating the complex processes controlling surfactant lipid homeostasis.

Transcriptional regulation of lipogenesis has been extensively studied in the liver and adipocytes. A number of TFs have been identified controlling the expression of lipogenic enzymes and genes in the lipogenic pathways including Sterol Regulatory Element Binding Protein (SREBP) isoforms, CCAAT-enhancer binding protein (C/EBP) isoforms, nuclear hormone receptors (NR1H2 and NR1H3) and peroxisome proliferator activated receptors (PPAR) [3-7]. SREBP has two genes (Srebf1 and 2) encoding for three protein isoforms, SREBP-1a, SREBP-1c and SREBP-2. SREBPs are synthesized as inactive precursors and activated by proteolysis in the Golgi apparatus. SREBP-2 primarily activates cholesterol biosynthetic genes whereas SREBP-1c predominantly activates genes involved in fatty acid production [4]. The C/EBPs belong to the basic-leucine zipper class of TFs. Six isoforms have been identified; all of which act as homo-or heterodimers via highly conserved bZIP domain [8]. The involvement of C/EBPs in lipogenesis is strongly supported by both *in vitro *and *in vivo *data. In adipocytes, C/EBPα, SREBP-1c and PPARγ induce fatty acid biosynthesis, but only C/EBPα is essential [9].

Lung maturation is highly dependent on the differentiation and function of the respiratory epithelium that, in turn, produces pulmonary surfactant lipids and proteins. Studies from the conditional deletion or mutation of specific genes have lead to the identification of several TFs in lung epithelium that are crucial to lung maturation and respiratory adaptation, include TTF-1, FOXA2 and C/EBPα. TTF-1 binds to the promoters of lung specific genes such as *Sftpa, Sftpb, Sftpc, Sftpd *and *Scgb1a1 *and increases their expression [10,11]. The deletion of *Foxa2 *or *Cebpa *from lung epithelial cells resulted in the lack of surfactant lipids and proteins, lack of appropriate differentiation of type I and II cells and absence of lamellar body formation, indicating delayed peripheral lung maturation [12,13]. Comparative microarray analysis show that although these TFs bind to distinct cis-elements in the promoter region of target genes, they are able to influence the expression of many common targets involved in surfactant proteins and lipid biosynthesis (e.g, *Abca3, Scd1, Pon1, Sftpa, Sftpb, Sftpc *and *Sftpd*), fluid and solute transport (e.g., *Aqp5, Scnn1g, Slc34a2*) and innate host defense (e.g., *Lys, Sftpa, Sftpd and Scgb1a1*), suggesting that *Foxa2, CEBPα *and *Titf1 *may share common transcription network regulating perinatal lung maturation and postnatal adaptation [12-15]. The majority of information regarding the role of SREBP has been focused to cholesterol and fatty acid metabolism in tissues such as liver and adipose [4,16,17]. SREBP-1c is expressed in the developing lung, where its expression increases during late gestation, concomitantly with the perinatal increases in surfactant lipid synthesis and the induction of genes critical for surfactant function [18,19]. SREBP activates CTP:phosphocholine cytidylyltransferase, the rate-limiting enzyme for phosphatidylcholine synthesis and increases surfactant phosphatidylcholine synthesis in the mouse lung [20-22]. These data strongly support the notion that in lung, SREBP may play an important role in surfactant and phospholipid homeostasis.

A fundamental challenge in the "post genomic era" is to decode transcriptional networks that direct intricate patterns of gene expression in complex organisms. In the lung, how TFs interact with each other and signaling molecules to regulate groups of gene targets mediating distinct but integrated aspects of cell or organ function, and how lipid homeostasis is integrated with maturation of type II epithelial cells remain unclear. It is highly likely that surfactant lipid homeostasis is controlled by complex interactions among transcriptional networks that integrate distinct but interrelated aspects of alveolar cell biology, e.g., lung maturation, host defense and surfactant function. Several strategies have been devised to decipher regulatory components and networks, each is partially successful and none is without limitations. Microarray analysis reveals mRNAs that change significantly in expression, but fails to assign these changes to biological events. The GO annotation and literature mining enable the association of genes with biological processes and pathways, but are limited to current knowledge. TF-TG correlation takes into account that expression profiles of TFs and their targets are often correlated and genes with highly correlated profiles are likely to be regulated by the same TF(s). In some instances, however, TFs regulate their targets, not by changing their own expression, but by post-transcriptional mechanisms such as transcript stability, binding site accessibility, interaction with tissue-specific co-factors or chromatin structures [23,24]. Promoter analysis seeking conserved or common TFBSs in promoters of co-expressed genes can identify the potential cis-elements, but may not inherently identify the binding TF or its role in transcription; moreover, this analysis is often associated with high numbers of false positive predictions due to the short and degenerate nature of many TFBS motifs. In the present study, we sought biological consistency and comprehensiveness by using a systems approach to integrate analytic results from independent and complementary resources, including gene expression profiling, protein interaction, functional annotation, promoter and literature mining, to develop a map of genetic networks regulating lung lipogenesis and surfactant homeostasis that are critical for lung function, focusing on the roles of key TFs in the network.

## Results and Discussion

We retrieved microarray data from a lung specific gene expression database that measures lung mRNA responses to genetic modification of various genes important to lung development and function (see "Data collection, processing and storage"). Total of 194 mRNA microarray samples from 27 distinct mouse models were used in this study (Table 1).

**Table 1:** Microarray Data Used In This Study

Array Name	Investigator	Mouse Model	Reference
CEBPA_KO	Ikegami	*Cebpa *^Δ/Δ ^mice: (tetO)_7_CMV-Cre^-/tg^/SP-C-rtTA^-/tg^/*Cebpa*^*flox/flox*^, E18.5	Martis, et al. 2006

CNB	Dave	*Cnb *^Δ/Δ ^mice: *CCSP-rtTA/(tetO)*_*7*_*CMV-Cre/Cnb1*^*flox/flox*^	Dave, et al. 2006

CTNNB1_ACT	Mucenski	Catnb^Δ (ex3) ^mice: *CCSP-rtTA*^+/tg or tg/tg^, *(tetO)*_*7*_*CMV-Cre*^+/tg or tg/tg^, *Catnb*^+/Δ (ex3)^	Mucenski, et al. 2005

CTNNB1_KO	Mucenski	*SP-C-rtTA*^+/*tg*^, (*tetO*)^*7*^*-CMV-Cre*^+/*tg or tg*/*tg*^, β-*catenin*^*flx*/*flx*^	Mucenski, et al. 2003

Cyclopamine_ Effect	Shannon	Lung explant culture treated with Cyclopamine for 1-3 days	

D508	Whitsett	*CFTR*^Δ508 ^mice: FABP-*hCFTR*^+/-^/*mCftr*^-/-^/SP-C-Δ508*CFTR*^+/+^	Xu, et al. 2006

FGF18_OE	Whitsett	*SP-C-rtTA and (teto)_7_CMV-FGF-18*	Whitsett, et al. 2002

Fgfr2IIIb	Perl	*SP-C-rtTA and (teto)*_*7*_*CMV-Fgfr2IIIb*^*flx*/*flx*^*; *E11.5-13.5 lung	Perl, et al. 2003

FoxA2_KO	Whitsett	*Foxa2 *^Δ/Δ ^mice: SPC-*rtTA*^-*/tg*^/(tetO)_7_*Cre*^-*/tg*^/*Foxa2*^*LoxP/LoxP*^; E18.5	Wan, et al. 2004

FoxaDKO	Whitsett	*Foxa2 *^Δ/Δ^, *Foxa1*^-/- ^mice: *Foxa1*^-/-^/SPC-*rtTA*^-*/tg*^/(tetO)_7_*Cre*^-*/tg*^/*Foxa2*^*LoxP/LoxP *^; E14.5	Wan, et al. 2005

FoxM1_KO	Whitsett	*Foxm1-/- *mice; E18.5	Wang, et al.

HIF1KO	Shannon	*Hif1 *^Δ/Δ ^mice: SPC-*rtTA*^-*/tg*^/(tetO)_7_*Cre*^-*/tg*^/*Hif1*^*flx/flx*^; PND1	

LAL	Yan	*Lal-/- *mice, 4month	Lian, et al. 2004

MIA	Shannon	*tetO7-Cre/SPC-rtTA/Mia1*, E18.5	Lin, et al. 2008

SHH12.5	Shannon	*Shh-/- *mice; E12.5	

SHH13.5	Whitsett	*Shh *^Δ/Δ ^mice: *SP-C-rtTA*^*tg*^*/(tetO)*_*7*_*CMV-Cre*^*tg/tg*^*/Shh*^*flx/flx*^; E13.5	Miller, et al. 2004

SHH18.5	Whitsett	*Shh *^Δ/Δ ^mice: *SP-C-rtTA*^*tg*^*/(tetO)*_*7*_*CMV-Cre*^*tg/tg*^*/Shh*^*flx/flx*^; E18.5	Miller, et al. 2004

SPA_KO	Levine	alveolar macrophage from *Sftpa -/- *mice	

SPC_2M	Glasser	*Sftpc-/- *mice; 2month	Glasser, et al. 2003

SPC_PND1	Glasser	*Sftpc-/- *mice, PND1	Glasser, et al. 2008

SPC_typeII	Glasser	Isolated typeII cells from *Sftpc -/- *mice	Glasser, et al. 2003

SPD_AM	Whitsett	Isolated alveolar macrophage from *Sftpd-/- *mice	Zhang, et al. 2006

SPD_typeII	Ikegami	isolated typeII cell from *Sftpd -/- *mice	Korfhagen, et al. 1998

Stat3_tyII	Ikegami	Type II cells from *TetO7Cre/SPC-rtTA/Stat3*^*flox/flox*^, 7 week.	Xu, et al. 2007

SU5402	Shannon	Lung explant culture treated with 0.1% DMSO or SU5402, E12.5	Metzger, et al. 2007

TTF1_Lung	Whitsett	*Titf1*^PM/PM ^mice:*Titf1 *phosphorylation mutant, E18.5 lung	DeFelice, et al. 2003

TTF1_Thyroid	Whitsett	*Titf1*^PM/PM ^mice:Titf1 phosphorylation mutant, E18.5 thyroid	

### Clustering and functional classification revealed three lipid enriched gene clusters

Cluster analysis grouped 1498 genes that significantly changed in response to various gene perturbations into 29 clusters (Additional file 1). Genes in each cluster were further classified according to GO classification by Biological Processes to test the biological relevance of each cluster. The criteria for an enriched functional class were P < 0.01 in Fisher Exact Test, the functional term being shared by more than 20% of the genes in the cluster. Most clusters (26/29) had enriched functional classes according to the criteria, indicating their functional coherence within each cluster (Table 2).

**Table 2:** Functional Classification of Gene Clusters

BioProcess	Clusters
Carbohydrate/organic acid metabolism	23, 24

cell adhesion	1,3,23,24

cell cycle	14,15,16

cell differentiation	1,10

cell migration/motility	3

defense response	10,20,21,22

development	1,3,10,19,23,27,28

DNA metabolism/replication	14,15

localization/transport	1, 3, 10,28

lipid metabolism	1, 2, 23,28

metabolism	5,6,8,9,12,13,14,15,16,17,23,24,28

morphogenesis	1,23,24,27,28

negative regulation of biological process	13,23,24

Regulation (Transcription/signaling)	5,6,9,11,14,15,16,18, 19, 23, 24

protein modification	12

regulation of cell size	24

RNA splicing	7

cytoskeleton organization and biogenesis	19, 24

blood vessel development	3,19

Clusters listed in Additional file 1 were subject to Gene Ontology analysis http://david.abcc.ncifcrf.gov/ to determine the extent of enrichment of biological function among genes in each cluster. Clusters sharing biological functions were grouped together according to the function. Shown in the table are enriched functional classes with enrichment p-value < 0.01 and shared by more than 20% of the genes in the cluster.

In the present study, we sought to identify the transcription networks regulating perinatal surfactant lipid homeostasis. "Lipid biosynthesis/metabolism/transport" was enriched in 4 out of 29 clusters and SREBP was a member in three of the clusters. We chose to focus on three SREBP related lipid clusters (C1, C2 and C28) for compactness and simplicity of the network (C23 was not included since SREBP was not in the cluster and lipid metabolism was not the predominant functional class of this cluster).

In addition to the commonly enriched functions, i.e. "lipid biosynthesis and metabolism", each cluster has its uniquely enriched functionality (Table 3). Cluster 1 is functionally enriched in "lung" and "vascular" development, with corresponding mouse phenotypes that include "abnormal vascular development, alveolar morphology and respiratory mechanics". Membrane/Insoluble fraction is the most enriched cellular component in cluster 1. Cluster 2 is the smallest cluster among the three and is enriched for "lipid metabolism and lipid transport". Mouse phenotypes associated with the cluster 2 include "abnormal respiratory alveolar morphology and abnormal lipid homeostasis". "Endoplasmic reticulum (ER)" is the most enriched cellular component in this gene cluster. Tissue distribution analysis indicated that the expression of genes in this cluster is most abundantly expressed in the lung. These functional annotations aligned well with the fact that surfactant lipid and proteins are synthesized and assembled in the ER of alveolar type II cells. Cluster 28 is functionally enriched in "lipid metabolism" and "response to external/chemical stimulus", the corresponding mouse phenotype being "abnormal blood chemistry and alveolar morphology". Overall, the functional classifications indicate that lung lipid metabolism is closely associated with lung development and is required for various stress responses.

**Table 3:** Clusters Feature Comparison

Cluster name	Gene Number	Function and Process	Mouse Phenotype	Cell Components
C1	313	Lipid biosynthesis; Morphogenesis; Differentiation; Proliferation; Lung and respiratory tube development; Vascular development	Abnormal vasculature development; Abnormal cardiovascular physiology; Abnormal alveolar morphology; Abnormal respiratory mechanics	Insoluble fraction; Membrane fraction

C2	54	Lipid Metabolism; Lipid Transport	Abnormal respiratory alveolar morphology; Abnormal lipid homeostasis	Endoplasmic reticulum

C28	205	Response to external stimulus; Lipid metabolic process	Abnormal blood chemistry	Insoluble fraction; Integral to plasma membrane

Genes from each cluster were subject to gene set enrichment analysis to identify enriched functions and processes, mouse phenotypes and cell components http://toppgene.cchmc.org/.

### Identification of commonly enriched TFBS

**Figure 1:**
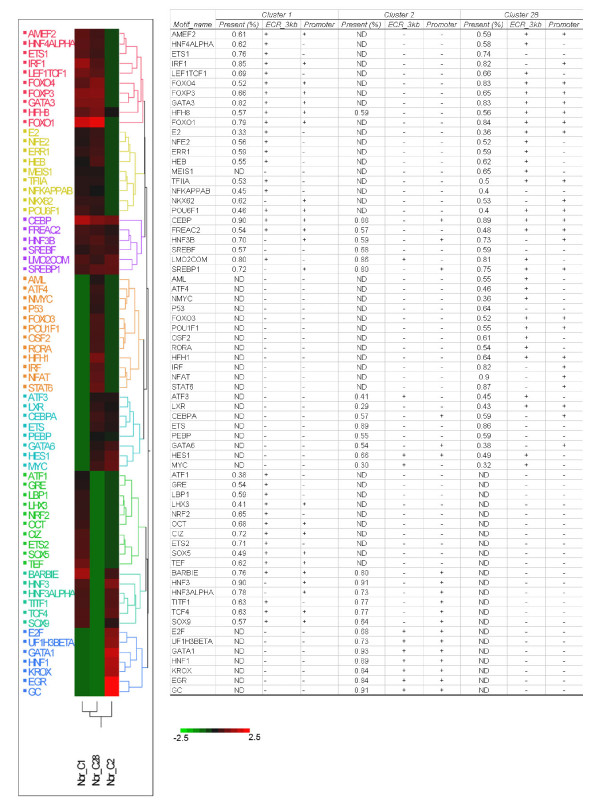
**Identification of over-represented TFBSs in each gene cluster**. Upstream genomic sequence (3 kb) was searched for TFBS in evolutionarily conserved regions (ECR) that are over-represented in a gene cluster. Proximal promoter regions (1.2 kb) were searched for over-represented TFBS in the cluster. We also determined the over-represented TFBS frequency in the proximal promoter region for each gene in the cluster. The relative importance of a TFBS was determined by the average ranking order of ECR, prompter and frequency analysis and normalized to -2.5 to 2.5. A heatmap was generated based on the normalized relative importance of TFBSs. ND: Frequency was not determined if the TFBS was not enriched in the promoter region of the gene cluster compared to all promoters in the mouse genome used as the background set (p-value > 0.05).

In general, transcriptional regulation is mediated by the binding of TF or their partners to specific binding sites (TFBS) in the regulatory regions of the target genes (TG). The TFBSs are often located in close proximity to the transcription start site (TSS), but sometimes can be located at more remote locations [25-27]. It is assumed that functional TFBS are subject to greater selective pressure, and therefore will be evolutionarily conserved across species [28-30]. To identify over-represented TFBSs in a given cluster, we took three approaches. First, we searched 3 kb upstream genomic sequence for TFBS in the evolutionarily conserved regions (ECR) that are over-represented in a gene cluster [28,31]. We then searched proximal promoter regions (1.2 kb) for over-represented TFBS in the cluster [32]. We also determined the over-represented TFBS frequency in the proximal promoter region for each gene in the cluster. The relative importance of a TFBS was determined by the average ranking order of the ECR, promoter and frequency analysis. The results are summarized in Figure 1. TFBS for CEBP (CCAAT/Enhancer Binding Protein Family), HNF3B (FOXA2) and SREBP (SREBF1/2) are common to all three clusters, likely indicating the universal roles of these factors in lung lipid metabolism. TFBS for TTF1, HNF3 (FOXA1/2), TCF4, SOX9 and BARBIE (barbiturate-inducible element) were commonly enriched in cluster 1 and 2 genes. The enrichment of this group of TFBS in cluster 1 and 2 gene promoters may indicate their related roles in lung development and morphogenesis. In addition to commonly enriched TFBS among the clusters, we identified TFBS uniquely enriched for each cluster. For example, CIZ (Cas-associated zinc finger protein), OCT (POU2F1) and ETS2 were unique to C1 genes; HNF1 and EGR1 were unique to C2 genes; NFAT and STAT6 were unique to C28 genes. This was consistent with the finding that the three clusters have shared as well as unique functionalities.

### Determination of TF-TG functional similarity and expression correlation

It is assumed that genes share similar annotations are likely to be involved in similar biological processes. We used kappa statistics to quantitatively measure the degree of agreement how TF-TG sharing annotation terms [33]. Kappa result ranges from 0 to 1. The higher the value of Kappa, the stronger the agreement is. The annotation terms are downloaded from DAVID knowledgebase http://david.abcc.ncifcrf.gov/. We calculated the kappa similarity between the enriched TFs of a given cluster (determined via promoter analysis) and genes in the same cluster. Table 4 lists top ranked genes according to their functional similarity (kappa) to that of *Srebf1 *and *Cebpα*.

**Table 4:** TF-TG functional similarity and expression correlation (CEBPA and SREBP)

CEBPA				SREBP			
**Rank by Kappa Similarity**	**Known Targets**	**Rank by correlation**	**Known Targets**	**Rank by Kappa Similarity**	**known Targets**	**Rank by Correlation**	**Known Targets**

Cebpa	Kyrmizi et al. 2006	Cebpa	Kyrmizi et al. 2006	Srebf1	She et al. 2005	Srebf1	She et al. 2005

Foxf1a	Kim et al, 2005	S100 g		Mtdh		Lipg	Reed et al. 2008

Foxa1		Lpcat1		Supt16h		Wdr81	

Ets1	Lefterova et al. 2008	Sftpb	Martis et al. 2006	Id2		6330416G13Rik	

Sox7		Dlk1	Shimomura et al. 1998	Ebf1		Abca3	

Foxa2	Martis et al 2006	Serpinb6b		Elf5		Wars	Reed et al. 2008

Wwtr1		Timp3	Lefterova et al. 2008	Ankib1		Lyzs	

Elf5		Edil3		Fah		Serpinf1	

Smad5		Abca3		Fli1		Dhcr7	Reed et al. 2008

Tbx4		3110001I20Rik		Soat1	Farrell, et al. 2005	Siva1	

Fli1		Bex2		Ahr	Iwano et al. 2005	Ndst1	

Ahr		Tspan11		Cdkn2b		Cds2	

Etv5		Vsnl1		Foxo3		Bcl6b	

Id2	Tavor et al. 2003	Cd38		Sox7		Matn4	

Runx1t1	Rochford et al 2004	1190002N15Rik		Mid1ip1		Ier3	

Mef2c		Pard6b		Cbfa2t3		Scd1	Horton et al. 2002

Ebf1	Jimenez, et al. 2007	Emp2		Mef2c		Hck	

Klf7		Id2	Tavor et al. 2003	Myb		Dag1	

Prdm1		Kit		Zfx		Bcl2a1a	

Jun	Rangatia et al. 2002	Mme		Acsl4		Sox7	

Utp11l		B3gnt2		Cebpa	Pedersen et al. 2007	Ahr	Iwano et al. 2005

Tcfcp2l1		Ndst1		Dhcr7		Rab6b	

Cbfa2t3		Lyz1	Lefterova et al. 2008	Etv5		Slc1a5	

Fos	Cammenga et al. 2003	Lyz	Lefterova et al. 2008	Foxa1		Slc34a2	

Stat3	Numata et al. 2005	Syne2		Foxa2		Enpp2	

Sox2		Tgoln1		Rab2		Hdc	Ai et al. 2006

Myb	Verbeek, et al. 1999	Klf7		Runx1t1		Sftpb	

Srebf1	Le et al. 2002	Atp6v1b2		Tbx4		Kdr	

Klf9		Me1	Lefterova et al. 2008	Tcfcp2l1		Tsn	

Foxo3a		Tcfcp2l1		Upk3b		Rtkn2	

Cdkn2b		Rtkn2		Foxf1a		Zdhhc14	

Ankib1		Krt19		Sox2		Dtna	

Fah		Slc34a2		Stat3		Lphn3	

Mapk14	Kumar et al. 2003	Prdx6		Aytl2		Lpcat1	

Cyp4v3		Fabp5		Ets1		Scd2	Tabor et al. 1999

Elovl1		Ier3		Exosc7		Emp2	

Qk	Lefterova et al. 2008	Scd1	Christy et al. 1989	Elovl1		Hc	

Rcan1		Cd55		Fos		Cyp4v3	

Exosc7		Exosc7		Ggcx		Mid1ip1	

Gadd45g		Kdr		Klf9		Lyz	

Correlation: the expression profile similarities between TF and genes in the same cluster were calculated using Pearson Correlation and ranked accordingly from high to low based on the correlation coefficient. The top 40 genes with the highest correlation with Cebpa and Srebf1 are listed in Table 4.

Kappa similarity was calculated to estimate functional similarity between TF and genes based on the shared annotation terms. TF-TG functional association were ranked from high to low based on the Kappa value. The top 40 genes sharing the highest functional annotations with Cebpa and Srebf1 are listed in Table 4.

We collected the positive TF-TG relationships from Ingenuity knowledge base (Ingenuity), Transfac 11.3 (Biobase), Eldorado (Genomatix) and PubMed. References for the known TF-TG relationships are listed in the table.

Expression profiles of transcriptional regulators and their targets are correlated in many cases, and genes regulated by the same regulators are likely to be co-expressed [34-37]. We considered TFs in each cluster as potential regulators of the genes in the same cluster. We determined the TF-TG correlations using Pearson correlation. *Srebf1 *and *Cebpa *expression profiles correlated well with many of the genes in the lipid clusters across various experimental conditions, there were 50 genes correlated with *Srebf1 *and 60 genes correlated with *Cebpa *with a correlation coefficient ≥0.5. Table 4 lists genes whose mRNA expression was strongly correlated with that of *Srebf1 *and *Cebpα *in the rank order of the Pearson product-moment correlation coefficient. As indicated in the Table 4, regulation of a number of the top ranked genes by *Srebf1 *and *Cebpα *was experimentally confirmed through literature search, indicating TF-TG functional similarity and expression correlation can be useful features for TF-TG prediction.

TF-TG functional similarity measure is limited by known annotations for a given gene. Likewise, correlation does not always hold true. For example, previous studies using lung selective deletion of *Foxa2 *in the mouse demonstrated that *Foxa2 *is critical for lung maturation and is involved in the expression regulation of genes in surfactant lipid synthesis [13]. The promoter and gene ontology analysis also indicate that Foxa2 is an important regulator in the mouse lung lipid network. Foxa2 mRNA levels were poorly correlated with genes in the lipid clusters, there were only 5 genes that correlated with *Foxa2 *with a correlation coefficient ≥0.5. We confirmed by qRT-PCR that *Foxa2 *mRNA expression levels do not substantially change during lung maturation (data not shown). TFs can regulate their targets independently of their own levels of expression, for example by changing chromatin structure, histone-modification states, nucleosome positions in vivo, phosphorylation status, and binding site accessibility with other partners [23,24]. In other words, a positive correlation between TF and TG provides useful evidence for a regulatory relationship; a poor expression correlation does not necessarily indicate there is no relationship between a given TF-TG pair. Our predication is based on the combined evidence from mRNA expression correlation with promoter profiles and gene ontology similarity; the latter two methods do not require expression correlation.

### Prediction of Gene Regulatory Interactions via Data Integration

We then predicted TF-TG interaction based on the integration of evidence from TF-TG correlations, promoter TFBS information, TF-TG kappa similarity and TF-TG interaction evidence. An integrative scoring function was developed to rank the possibility of TF-TG relationship, and to prioritize and associate each target with its potential regulators (detail see METHODS section). Based on these regulatory relationships, we constructed a lung lipid regulatory network. Using the cut off confidence score of 0.5, the overall connectivity of each TF was calculated and summarized in Table 5. HNF3, ETSF, SREBP, CEBP, GATA and IRFF were the most common TFBSs across the three lipid clusters with the highest connectivity in the network. Using this method, we linked the TFs to their potential target genes in three lipid clusters in the order of confidence score (Additional files 2, 3, 4). The TFBS of SREBP, HNF3 and CEBP are commonly enriched in all three lipid related clusters and share many downstream targets. Additional files 5, 6, 7 listed top ranked potential targets for SREBP, CEBP and HNF3 according to the confidence score from the integrative analysis of three lipid related clusters. Within the top 100 predicted targets for CEBP, SREBP and HNF3, 49 were common between SREBP and CEBP, 44 were common between CEBP and HNF3, and 35 were common between SREBP and HNF3; suggesting complex crosstalk and interactions among CEBP, SREBP and HNF3 in the proposed lung lipid network.

**Table 5:** Summary of TF connectivity

TFBS	Total Connectivity	C1	C2	C28	TF in Lung
CEBP	447	238	51	158	Cebpa, Cebpb, Cebpd, Cebpg

IRFF	404	239	0	165	Irf1, Irf2, Irf3, Irf5, Irf7

HNF3	359	228	51	80	Foxa1, Foxa2

GATA	358	218	44	96	Gata6, Gata1

ETSF	344	172	14	158	Ets1, Ets2, Etv5, Nfe2l2, Elf2

SREB	312	162	43	107	Srebf1

FOXO	268	151	0	117	Foxo1, Foxo4, Foxo3a

FKHD	213	95	25	93	Foxf2, Foxc1

HAND	201	94	0	107	Lmo2

STAT	182	0	0	182	Stat6, Stat3

MEF2	176	110	0	66	Mef2a

NFAT	169	0	0	169	Ilf3, Nfatc3

CP2F	168	83	0	85	Atf4, Tcfcp2, Atf3, Atf1

NFKB	166	78	0	88	Nfkb1

EREF	165	113	0	52	Esrra

LEFF	150	97	0	53	Lef1

HFH	134	75	19	40	Foxf1a, Foxi1

PARF	134	134	0	0	Tef, Tead1

AP1R	129	60	0	69	Nfe2

LEFF	121	87	34	0	Tcf4

CIZF	117	117	0	0	Znf384

HAND	113	43	0	70	Tcf12

BARBIE	111	98	13	0	Unknown

NKXH	106	68	38	0	Nkx2-1

SORY	104	93	11	0	Sox5, Sox9

NR2F	98	37	20	41	Hnf4a, Nr2f1, Nr2f2

OCT	92	92	0	0	Pou2f1, Pou6f2

CREB	79	0	10	69	Creb1

MYOD	74	0	0	74	Myog

NKX7	62	39	0	23	Nkx6-2

EBOX	59	0	21	38	Tcf4, Max

P53F	57	0	0	57	Trp53

RORA	57	0	0	57	Rora

HAML	54	0	0	54	Runx2, Pebp1

RXRF	54	0	0	54	Nr1h2

GREF	48	48	0	0	Nr3c1

BRN5	46	25	0	21	Pou6f1

HESF	46	0	20	26	Hes1

EGRF	45	0	45	0	Egr1, Wt1

HOXH	44	0	0	44	Meis1

SPIF	39	0	39	0	Klf11

HNF1	38	0	38	0	Hnf1a, Hnf1b, Hmbox1

E2FF	33	0	33	0	E2f1, E2f2, E2f3, E2f4, E2f5, E2f7

SMAD	23	0	23	0	Smad4

ZBPF	23	0	23	0	Zfp219

NKX6	22	22	0	0	Nkx6-1

LXHF	21	21	0	0	Lxh3

AP2F	19	0	19	0	Tcfap2c

PTBP	19	0	19	0	Tbp

GLIF	16	0	16	0	Zic2

BCDF	15	0	15	0	Crx

SPZ1	11	0	11	0	Spz1

PAX2	10	0	10	0	Pax2

MTF1	9	0	9	0	Mtf1

ZF5F	8	0	8	0	Zfp161

We calculated the confidence score based on the integrative evidence of TF-TG relationship. Using the cut off confidence score of 0.5, the overall connectivity of each TF to its potential TGs within three clusters were calculated and summarized in Table 5. The corresponding TFs expressed in lung were also listed.

This method enables identification of genes of interest and their regulators in rank order of their confidence level (Table 6). For example, *Abca3 *is predicted to be regulated by TFs in the order of SREBP1, HNF3 (FOXA1/2), TTF1, EGR (EGR1), E2F (multiple family members) and CEBPA. ABCA3 is a known phosphatidylcholine transporter and plays an essential role in pulmonary surfactant lipid metabolism and lamellar body biogenesis [38,39]. *ABCA3 *mutations are associated with surfactant deficiency and fatal respiratory distress syndrome [40-42]. Our previous studies showed that *Abca3 *gene expression was regulated by SREBP, CEBPA and FOXA2 [12,13,43]. *Abca3 *promoter activity was regulated by both lung selective TFs including TTF1, CEBPA and FOXA2 and the lipogenic TF SREBP1. The direct binding of SREBP1c to *Abca3 *promoter was confirmed by ChIP assay [44]. Thus *Abca3 *expression is regulated by both cis-acting cassettes, providing a mechanism by which surfactant and lipid homeostasis may be integrated at the transcriptional level [44]. In addition to known regulators, our model predicts EGR and E2F as potential important regulators for *Abca3 *expression. ELOVL1 encodes a microsomal enzyme involved in tissue-specific synthesis of very long chain fatty acids and sphingolipids [45,46]. Little is known about *Elovl1 *expression regulation other than that its mRNA expression is correlated with SREBP1 in brown adipocytes [47]. *Elovl1 *was grouped in Clusters 1 and 2, together with *Abca3 *and our analysis predicts its control by SREBP, CEBP, HNF3, TTF1 and TCF4, sharing similar regulation with *Abca3*. *Slc34a2 *encodes Na(+)/Pi cotransporter 2B (NPT2B), is expressed most strongly in lung and only in apical membrane of alveolar type II cells, the cells that produce and secrete surfactant. Because of this localization, it was proposed that the function of the gene product is to take up phosphate from the alveolar fluid [48,49]. Mutations in *SLC34A2 *cause pulmonary alveolar microlithiasis [48,50]. We utilized transient transfection promoter assays and confirmed the activation of *Elovl1 *and *Slc34a2 *transcription by both SREBP1 and CEBPA (see data validation section). DLK1 encodes an EGF like homeotic transmembrane protein that acts as a negative regulator of Notch1 and adipocyte differentiation [51,52]. Our analysis predicts its control by CEBP, HNF3, SREBP1, EGR1, HNF1 and GATA1. Both *Elovl1 *and *Dlk1 *are highly enriched in alveolar type II cells. Based on the present model, we hypothesize that genes such as *Slc34a2, Dlk1 *and *Elovl1 *may share similar transcription regulation with *Abca3 *in the lung where they are likely to influence surfactant metabolism.

**Table 6:** Selected Genes and their potential regulators in rank order

Gene TF Rank	ELOVL1	SLC34A2	SOAT1	ZDHHC3	LPCAT1	STARD4	DLK1	PRDX6	ABCA3
*1*	SREBP (Srebf1/2)	CEBP (Cebpa/b/g)	SREBP1 (Srebf1/2)	SREBP1 (Srebf1)	CEBP (Cebpa/b/g)	SREBP1 (Srebf1)	CEBP (Cebpa/b/g)	CEBP (Cebpa/b/g)	SREBP1 (Srebf1)

2	CEBP (Cebpa/b/g)	ETS1 (Ets1)	ZIC2 (Zic2)	GATA (Gata6)	SREBP1 (Srebf1)	CEBP (Cebpa/b/g)	HNF3 (Foxa1/2)	SREBP (Srebf1/2)	HNF3 (Foxa1/2)

3	HNF3 (Foxa1/2)	STAT6 (Stat6)	CEBP (Cebpa/b/g)	HNF3 (Foxa1/2)	NFAT (Ilf3, Nfatc3)	ETS1 (Ets1)	KROX (Egr1)	NFKB (Nfkb1)	TTF1 (Nkx2-1)

4	TTF1 (Nkx2-1)	SREBP1 (Srebf1/2)	KROX (Egr1)	XFD1 (NP)	ETS1 (Ets1)	FOXP3 (NP)	SREBP1 (Srebf1)	ETS1 (Ets1)	EGR (Egr1)

5	TCF4 (Tcf4)	ERR1 (Esrra)	UF1H3B (Foxa1/2)	IRF1 (Irf1)	EGR (Egr1)	HNF3 (Foxa1/2)	HNF1 (Hnf1a/1b)	HNF3 (Foxa1/2)	KROX (Egr1)

6	NFKB (Nfkb1)	LMO2COM (Lmo2)	IRF1 (Irf1)	FREAC7 (NP)	STAT6 (Stat6)	ETS2 (Ets2)	TAXCREB (Creb1)	TTF1 (Nkx2-1)	E2F (E2f1-5)

7	ZIC2 (Zic2)	HEB (Tcf12)	LXR (Nr1h2)	TTF1 (Nkx2-1)	E2F (E2f1-5)	ATF1 (Atf1)	GATA1 (Gata1)	TCF4 (Tcf4)	CEBP (Cebpa/b/g)

8	SMAD4 (Smad4)	HNF3 (Foxa1/2)	LMO2COM (Lmo2)	EGR (Egr1)	GATA1 (Gata1)	HNF4A (Hnf4a)	ETS1 (Ets1)	NRF2 (Gabpa)	GATA (Gata6)

9	BARBIE (NP)	TATA (Tbp)	WT1 (Wt1)	ZF5 (Zfp161)	IRF1 (Irf1)	GATA3 (Gata6)	WT1 (Wt1)	SMAD4 (Smad4)	ZNF219 (Zfp219)

10	ETS2 (Ets2)	HNF4A (Hnf4a)	SMAD4 (Smad4)	AP2G (Tcfap2c)	AP2G (Tcfap2c)	ERR1 (Esrra)	STAT (Stat3)	GRE (Nr3c1)	WT1 (Wt1)

Genes were selected based on their functional relevance to surfactant biosynthesis/transport. For each gene of interest, its potential TF regulators were predicted in rank order of TF-TG confidence score and TF-TG relationships were supported by promoter assay. Top 10 TFBS and their corresponding TF in lung are listed under the selected genes.

### Construction of a lipid gene regulatory network in the mouse lung

**Figure 2:**
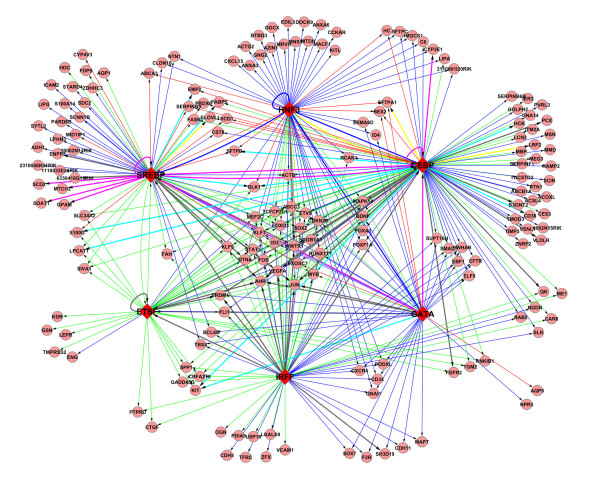
**Graphic representation of a subnetwork consisting of predicted TF-TG pairs with the highest connectivity**. The graphic representation of a subnetwork consisting of predicted TF-TG pairs with confidence cutoff as 0.60 and the top 6 TFs with the highest connectivity. SREBP, HNF3, ETSF, CEBP, GATA and IRFF were identified as regulatory hubs in this network. The network has 183 nodes and 386 links. Round nodes represent TGs, red diamond nodes represent TFs. Blue edges indicate the TF-TG predictions from C1, red edges for C2, green for C28, yellow for both C1 and C2, brown for both C1 and C28, light blue for both C2 and C28, and pink edges for TF-TG predication from C1, C2, and C28. The thickness of the edge corresponds to the frequency of the TF-TG prediction from all three clusters.

A lung "Lipid gene regulatory network" was generated by combining the predicted TF-TG relationships from the three clusters. In Figure 2, we show a sub-network consisting of the TFs with the highest connectivity (score ≥0.6, top 4.5%) among three gene clusters. SREBP, HNF3, ETSF, CEBP, GATA and IRFF are clear regulatory hubs in this network, these TFs are likely to be key regulators controlling surfactant lipid homeostasis in the lung via the regulation of genes within the three lipid-related clusters. The roles of several key TFs in the proposed network have been partially confirmed by previous studies from our group and others, including SREBP1, FOXA2, CEBPA, ETV5 and GATA6 [12,13,43,53-55]. IRF1 encodes interferon regulatory factor 1, a member of the interferon regulatory transcription factor family. The finding that IRF may serve as an important regulator in lung lipid homeostasis merits further experimental validation. The finding that previously experimentally validated transcriptional regulators of surfactant homeostasis were identified as key hubs in present unbiased network, strongly support the reliability of our proposed model.

**Figure 3:**
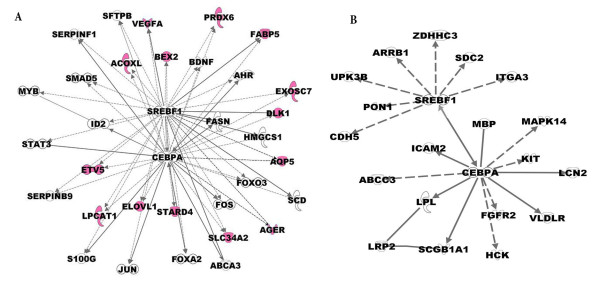
**Graphic representation of a CEBPA-SREBP centered sub-network**. The graphic representation of a CEBPA-SREBP centered sub-network, showing the potential connections between SREBP, CEBPA and their predicted gene targets. 3A represents top ranked common gene targets for CEBP and SREBP and 3B represents top ranked unique gene targets for CEBP or SREBP. Solid line represented literature-validated relationships and dotted lines represent predicted relationships. Known markers of lung maturation and function are highlighted in purple.

**Figure 4:**
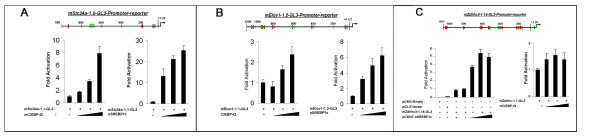
**Promoter reporter assay of predicted C/EBPA and SREBP targets in transient transfection of MLE-15 cells**. Schematic representation of the ≥1 kb *Slc34a2, Elovl1 and Zdhhc3 *promoter-luciferase constructs made in pGL3 reporter plasmids are depicted above the graphs. C/EBPα (green) and SREBP1c (red) represent consensus motifs on each mouse gene promoter. Transcription start sites are shown at +1 bp. The dose response effects of C/EBPα and SREBP1c expression after co-transfection with fixed amounts of the promoter-reporter constructs were assessed in MLE-15 cells, an immortalized mouse lung epithelial cell line, as measured by luciferase activity in 6-well plates. Values represent two independent experiments carried out in duplicate with means ± S.D. (*n *= 6).

Due to the complexity and modularity of the biological networks, we have focused on several important sub-networks. Figure 3 depicts the CEBPA-SREBP centered sub-network. 3A represents top ranked common gene targets for CEBP and SREBP and 3B represents top ranked unique gene targets for CEBP and SREBP. Many known markers of lung maturation and function, including SFTPB, ABCA3, AQP5, LPCAT SMAD5, ETV5 (Erm) and VEGFA are predicted to be co-regulated by SREBP and CEBPA. Further studies are needed to understand how the proposed interactions between SREBP and CEBPA control lung maturation. A subset of predicted targets whose regulation was unknown previously was experimentally confirmed by *in vitro *promoter reporter assays (Figure 4).

### Experimental validation of predicted TF-TG relationships

Network prediction was validated through promoter reporter assays, transgenic animal models and literature confirmation. Since the integrative analysis predicted SREBP, CEBP and HNF3 as key regulators in the lipid related transcription network in lung, we focused on these three TFs to validate the network predictions derived from the bioinformatics analysis.

Gene promoter assays were carried out on selective TF-TG pairs utilizing the following selection criteria: 1) confidence score, prioritizing top ranked gene targets of SREBP and CEBP, 2) tissue and cell specificity i.e. lung epithelial type II cell enrichment and subcellular location in endoplasmic reticulum or Golgi, 3) functional annotation that is lipid related and 4) originality, seeking novel targets with new function. Applying these criteria, we selected the first set of candidate genes likely modulating lipid homeostasis in the lung epithelial cells, including *Elovl1, Slc34a2 and Zdhhc3*, their functionality, expression and subcellular location as listed in Table 7. Figure 4 shows the promoter-reporter assays using C/EBPα and SREBP1c cotransfected with ~1 kb *Elovl1, Slc34a2 and Zdhhc3 *promoter-luciferase constructs in mouse lung epithelial cells (MLE-15)[56,57]. Consistent with our prediction (Table 6 and Additional files 2, 3, 4), CEBPA and SREBP1c activated *Elovl1 and Slc34a2 *promoters; *Zdhhc3 *was only regulated by SREBP1c but not by CEBPA. The functions of Elovl1 and Zdhhc3 in lung biology are unknown whereas *Scl34a2 *has recently been linked to alveolar microlithiasis[48,50].

**Table 7:** Functionality and subcellular location of selected genes

Symbol	Description	Expression & Subcellular Location	Function	Disease
**Elovl1**	Elongation of very long chain fatty acid protein1	Expressed in lung type II cells. Endoplasmic Reticulum Membrane	Tissue-specific synthesis of very long fatty acids and sphingolipids. May catalyze the conversion of beta-ketoacyl CoA to beta-hydroxyacyl CoA or Reduction of trans-2-enoyl CoA to the saturated acyl CoA derivative.	Parkinson's disease

**Slc34a2**	Solute carrier family 34 (sodium phosphate), Member 2	Apical Membrane of Type II cells	Actively transporting phosphate into cells via Na+ cotransport. May have a role in the synthesis of surfactant in lungs' alveoli.	pulmonary alveolar microlithiasis, ovarian cancer

**Soat1 (Acat)**	Sterol O-acyltransferase 1	Expressed in lung type II cells. Endoplasmic Reticulum Membrane	Catylyzes the formation of fatty acid-cholesterol esters. Plays a role in lipoprotein assembly and dietary cholesterol absorption.	atherosclerosis

**Zdhhc3 (Godz)**	Palmitoyltransferase Zinc finger DHHC domain-containing protein 3	Expressed in lung type II cells. Golgi apparatus	Palmitoyltransferase with broad specificity; membrane protein trafficking	

**Lpcat1 (Atyl2)**	Acyltransferase-like 2 Phosphonoformate immuno-associated protein 3	Expressed in lung type II cells. Endoplasmic Reticulum and Golgi Apparatus Membrane	Mediates the conversion of 1-acyl-sn-glycero-3-phosphocholine (LPC) into phosphatidylcholine (PC). May synthesize phosphatidylcholine in pulmonary surfactant, play a pivotal role in respiratory physiology.	migraines

**Stard4**	START domain-containing protein 4	Expressed in lung type II cells. Mitochondria.	May be involved in the intracellular transport of sterols or other lipids. May bind cholesterol or other sterols	Huntington's disease

**Dlk1 (DLK)**	Protein delta homolog 1	Expressed in lung type II cells. Membrane	May function in adipocyte differentiation and in neuroendocrine differentiation	lung cancer, bronchiolo-alveolar adenocarcinoma, blepharophimosis, obesity, neoplasia, hypertriglyceridemia

**Prdx6**	Peroxiredoxin 6	Expressed in lung type II cells. Cytoplasm, Lysosome, lung secretory organelles.	Involved in redox regulation of the cell. Can reduce H(2)O(2) and short chain organic, fatty acid, and phospholipid hydroperoxides. May play a role in the regulation of phospholipid turnover as well as in protection against oxidative injury	acute allergic pulmonary eosinophilia, asthma, follicular adenoma, Huntington's disease, neoplasia

**Abca3**	ATP-binding cassette, sub-family A (ABC1), member 3	Expressed in lung type II cells. Membrane	Plays an important role in the formation of pulmonary surfactant, probably by transporting lipids such as cholesterol	surfactant metabolism dysfunction, inflation, respiratory failure, atelectasis

Type II cell expression information is obtained from PBGE DB. Subcellular location is based on Gene Ontology http://amigo.geneontology.org/ DB and GeneCard http://www.genecards.org/. Protein function is based on Uniprot Knowledgebase http://www.uniprot.org/uniprot/. Disease information is based on the Ingenuity knowledgebase (Ingenuity) and Genecard http://www.genecards.org/.

Transgenic mice were used in conjunction with mRNA microarray to identify genes and processes regulated by TFs and signaling molecules. The correlation between the genomic response of selective TF perturbation using transgenic mouse models and the integrative prediction derived from the present study provide *in vivo *evidence for the predicted TF-TG regulatory relationships. We compared predicted SREBP, HNF3 and CEBP targets with the genes differentially expressed in the lung after selective deletion of *Scap *(SREBP cleavage-activating protein), *Foxa2 *and *Cebpa *from respiratory epithelial cells[43,58]. These three arrays were not included in previously array analysis and network development, therefore can be used as independent data for validation. Genes with high confidence score (score >0.55) were used as positive prediction, genes with low confidence score (score <0.45) were used as negative control. Based on the binomial probability calculation, predicted gene targets showed significant overlap with genes responsive to the deletion of the respective TFs *in vivo *(p-value for SREBP: 1.03E-08, p-value for FOXA2: 0.0037, p-value for CEBPA: 1.61E-05). Within the top 100 ranked candidate targets for CEBP (*Cebpa/b/g*), 35 mRNA were decreased in response to the *Cebpa *deletion in the lung *in vivo*. Likewise, within the top 100 ranked candidate targets for SREBP (*Srebf1/2*), 25 mRNAs decreased in response to the *Scap *deletion *in vivo*; and within the top 100 ranked candidate targets for HNF3 (*Foxa1/2*), 21 mRNAs were decreased in response to the *Foxa2 *deletion (Additional files 5, 6, 7). These results provide evidence that SREBP, HNF3 and CEBPA regulate the predicted gene targets expression in lung *in vivo*.

Literature mining provides another resource to validate computational predictions for the enriched TFs and their potential target genes in the three lipid clusters identified in the present study. We used MedScan Reader, a Natural Language Processing (NLP) text-mining tool [59] (Ariadne Genomics) to search the entire PubMed database. For each TF - TG pair, this algorithm extracts various types of regulatory mechanisms and the effects of regulation by recognizing different domain-specific named entities in the input text and extracting functional relationships among them. As indicated in Additional files 5, 6, 7, all experimentally confirmed SREBP targets were ranked in the top 5% of our predictions; all confirmed HNF3 targets were ranked in the top 10% of our predictions. In the case of CEBPA, all of the experimentally confirmed CEBPA targets were ranked within the top 30% of our prediction with the score >0.5, 86% of them were ranked in the top 10% of our prediction. CCAAT/enhancer binding proteins (C/EBP) include multiple family members that bind to CEBP binding sites with different affinities; that may influence the precision of the present prediction.

Taken together, the consistency of results from *in vitro *reporter assays, transgenic mice and literatures support the validity of the present approach and its potential utility for predicting important TF-TG relationships in the proposed transcription regulatory network.

### Biological implication of the lung lipid transcription networks

In the present study, we identified both general and context dependent regulators of lung lipid homeostasis related to pulmonary surfactant. The TFBS of SREBP, HNF3B and CEBP are commonly enriched in all three lipid related clusters and share many downstream targets. We hypothesize that SREBP, CEBP and HNF3 family of TFs form core regulatory modules to maintain surfactant production. Consistent with our model, previous studies demonstrated that the deficiency of hepatic C/EBP in leptin-deficient mouse leads to impaired SREBP signaling [60], C/EBPα and SREBP-1 form complexes in hepatocytes and synergistically regulate the transcription of lipogenesis associated genes such as *Acly *and *Acss2 *[6]. Recent work from Payne et al. [17] demonstrated that SREBP-1c is directly regulated by C/EBP factors during adipocyte differentiation (α, β and δ) and C/EBPα plays a critical role in regulating SREBP-1c in the later stages of adipogenesis (adipocyte maturation). In the lung, C/EBPα and SREBP-1c play important roles in alveolar type II cells lipogenesis [19]. FOXA2 interacts with C/EBPα in mouse liver [61], FOXA2 is necessary for normal expression of C/EBPα in embryonic mouse lung epithelial cells [12].

Core TFs may cooperate with other factors in a context dependent manner. In addition to "lipid metabolism", SREBP is associated with target genes involved in other related biological processes in cooperation with other TFs. TTF-1 (gene symbol: *Nkx2-1*) plays a central role at various stages of lung development, essential for lung cell differentiation, maturation and proliferation, and for the production of surfactant proteins. TTF-1 binds to the promoters of lung specific genes such as *Sftpa, Sftpb, Sftpc, Sftpd and Scgb1a1 *and increases their expression [10,15,53,55,62,63]. The effects of TTF-1 are likely mediated by its interactions with other TFs and co-activators, including WWTR1 (also known as TAZ [10]), GATA6 [55], RAR [64], NFATC3 [57] and NFI [65]. In the present study, TTF-1 is enriched in Clusters 1 and 2, sharing many targets with SREBP to control lipid and surfactant biosynthesis and transport (*Abca3, Prdx6, Sftpa1, Sftpb, Sftpc, Dlk1 and Elovl1*), Apoptosis (*Ahr, Bex2, Fli1, Id2, Mef2c and Runx1t1*), transcription regulation (*Ahr, Bcl6b, Cebpa, Elf5, Etv5, Foxa2, Jun, Sox7 and Wwtr1*) and respiratory disease (*Abca3, Aqp5, Cftr, Dlk1, Kdr, Prdx6, Sftpa, Sftpb, Sftpc and Slc34a2*). Among these, predicted targets such as CEBPA, FOXA2, WWTR1, JUN, ABCA3, SFTPA and SFTPB have been identified as interaction partners or transcriptional targets of TTF-1[12,44,63,66-68]; targets like AHR, CEBPA, ID2 and DLK1 have the same relationships with SREBP[69-73], but little information is available regarding combinatorial regulation of targets by multiple transcription factors.

EGR is uniquely enriched in Cluster 2 genes (lipid cluster). EGR-1 belongs to C2H2-type zinc-finger protein family and activates genes required for differentiation and mitogenesis. In lung, EGR-1 plays a key role in the pathogenesis of IL-13-induced inflammatory responses [74]. The role of EGR-1 in lipid metabolism is unknown. Present study identified a number of EGR and SREBP shared common targets that associated with lung disease or function (*Abca3, Aqp5, Foxa2, Cebpa, Kdr *and *Sftpb*), lipid metabolism (*Abca3, Soat1, Dlk1, Scd1, Scd2, Lpcat1 and Fabp5*), cell growth and proliferation (*Btg3, Dlk1, Emp2 and Pdia5*). Among these, *Scd1 and 2 *are known target of SREBF1 [75], their mRNA expression are also dependent on EGR2 [76]. SCD and FABP5 are known to play important roles in lung specific phospholipids/surfactant biosynthesis [19,77]. LPCAT1 encodes lysophosphatidylcholine acyltransferase catalyzing the conversion of lysophosphatidylcholine to phosphatidylcholine in the remodeling pathway of phatidylcholine biosynthesis [78]. LPCAT1 is highly expressed in lung type II cells and plays a critical role in regulating surfactant phospholipid/surfactant biosynthesis [79].

Known disease associated genes were identified through the present network analysis. As predicted in Figure 3, ABCA3, DLK1, VEGFA, AGER, SLC34A2 and surfactant proteins are co-regulated by SREBP and CEBPA. Deficiency or mutation of surfactant proteins and ABCA3 cause interstitial lung disease and respiratory failure [40,41,80], PRDX6 is associated with allergic pulmonary eosinophilia and asthma [81,82], DLK1 is associated with bronchiolo-alveolar adenocarcinoma and lung cancer [83], VEGFA (vascular endothelial growth factor A) and KDR (VEGFR, a member of VEGF receptor) play important roles in lung maturation [84] and pulmonary hypertension [85], AGER (advanced glycosylation end product-specific receptor, also known as RAGE) is associated with acute allergic pulmonary eosinophilia [81], and mutations of SLC34A2 cause pulmonary alveolar microlithiasis [50]. The finding that the present approach identified genes and processes associated with human lung disease indicates its potential utility for the discovery of new genes and biomarkers that may be useful in understanding the pathogenesis of lung disorders.

## Conclusions

We employed a systems biology approach to begin mapping a transcriptional network regulating surfactant homeostasis in the lung. We identified novel and known TFs, signaling molecules and potential target genes within the network. SREBP, CEBP, HNF3, ETS, GATA and IRF were identified as regulatory hubs with high connectivity. We propose that SREBP, HNF3B and CEBP form a common core regulatory module mediating surfactant lipid homeostasis. These key TFs likely interact with other TF partners to regulate lung growth (OCT and NFKB), differentiation and maturation (TTF1 and EGR1), pulmonary host defense and inflammatory responses (IRF, NFAT and STAT). The present study provides a systematic view and working model of a transcriptional network regulating the formation and metabolism of the pulmonary surfactant system.

The current approach also has several important limitations. The approach is unlikely to identify epigenetic, post-transcriptional and gene-environmental interactions that may play important roles in gene regulation [23,24]. Likewise, we have not emphasized the study of enzymatic transport activities of the many enzymes and proteins identified in the network. All these will be important for our long-term understanding of lung lipid homeostasis, but are beyond the scope of the present study.

## Methods

### Data Collection, processing and storage

We have developed a relational database to store, manage and maximally utilize gene expression profile data collected from multiple investigators in Cincinnati Children's Hospital Medical Center, Division of Pulmonary Biology. We analyzed 194 microarray samples from 27 independent microarray experiments in this study (Table 1). Data was normalized using the Robust Multichip Average model [86] from R/Bioconductor package. The detection of differential expression was preformed using unpaired two-group Student's t-test for mutant and control at the P value ≤ 0.05. Additional filters for positive candidate selection include a minimum of 1.5 fold change in absolute ratio and a minimum of 67% Present call by Affymetrix algorithm. We identified 1498 genes that significantly changed in response to the gene perturbations in at least 5 experimental conditions. The full gene set derived from mRNA profiling is listed in Additional file 1.

### Cluster analysis

Clustering is a powerful way to explore complex gene expression data by grouping them on the basis of similarity of their expression patterns. We compared methods among K-means, QT clustering and Fuzzy Heuristic Partition [87,88] in this study. Only Fuzzy Heuristic Partition allows genes to be assigned to more than one cluster with different degrees of membership. At a very stringent membership cutoff, most of the genes in each cluster were highly correlated across all experimental conditions. As the membership cut-off decreases, additional genes were assigned to the cluster based on their expression similarity on a subset of experimental conditions. This enables the identification of context-dependent regulation. We further clustered differentially expressed genes using Fuzzy clustering by local approximation of membership algorithm [87] with parameter setting -KNN: 7; Max App: 500; Membership Range: 35%. We evaluated the clustering performance based on its ability to produce biologically meaningful clusters using the Gene Ontology database as a common reference [89,90].

### Functional classification

After identifying co-expressed gene groups, we sought to identify the potential biological themes represented by these distinct gene sets. Such processes are helpful in assigning the functional linkage to gene groups and the evaluation of clustering quality. Genes in each cluster were uploaded to DAVID, a pre-compiled web-based functional annotation tool [91] for gene ontology analysis. For each GO term, a Fisher's exact test was used to compare the occurrence of the term in the list of interest and the rest of the genome as a reference list to identify over-represented functional categories in each gene list. For genes within a cluster, Kappa similarity was measured to estimate functional similarity between genes based on the number of shared annotation terms [33]. A TF-TG Kappa similarity matrix was created with each value ranging from 0 to 1, the higher the value of Kappa, the stronger the overall agreement in annotation terms.

### TF-TG Correlation

We consider TFs in a given cluster as "candidate regulators" of that cluster. The expression profile similarity between TF and genes in each cluster were calculated using Pearson Correlation and a TF-TG correlation matrix was generated with each value ranging from +1 to -1, indicating the perfect positive and negative correlation, respectively.

### Identification of common TFBS motif and module

Motif search is often associated with a large number of false positive predictions due to the short and degenerate nature of many TFBS motifs. Several approaches were used to reduce false positives and improve the prediction accuracy. 1) Apply comparative genomics: Genome RVista http://genome.lbl.gov/vista/ and DiRE http://dire.dcode.org were used to identify evolutionarily conserved regulatory elements that were over-represented in our co-expressed gene clusters [28,31,92]. Both use precompiled evolutionary conserved regions (ECR) via human and mouse whole genome alignment. The locations of putative TFBSs were precomputed for each genome using vertebrate position weighted matrices from TRANSFAC matrix library version 10.2. For Genome RVista, we chose conserved TFBSs located 3 kb upstream of transcription start site with the p-value cutoff at 0.005. For DiRE, we chose conserved TFBSs from the top three conserved ECRs (which can be located in intron, UTR or intergenic regions) and the promoter ECRs. Over-represented TBFSs from both programs were combined for further analysis. 2) Search for over-represented TFBSs in proximal promoter region: since the majority of functional TFBSs are found in the promoter region of eukaryotic genomes, cis-element over-representation (Clover) [93] was used to determine the conserved TFBSs that were over-or under-represented in the given promoter set. 3) Search for Cluster and composite of TFBSs: Since it is known that TFBS are not evenly distributed, finding motif peaks within the promoter region is likely to indicate functional regulatory regions. Cluster-Buster, a Hidden Markov Model based method [93] was used to identify clusters of pre-specified motifs in a given gene cluster. Perl scripts were used to extract common composite sites from the motif clusters identified by Cluster-Buster algorithm. For approaches in 2) and 3), we used proximal promoter sequences of genes in the cluster of interest (1 kb up stream and 200 bp downstream of TSS, Ensembl release, version 52). We used MousePromoters_v19 from Ensembl release 19.32 as the background set, which contains 20,028 mouse promoters of the same region. 4) Both TRANSCompel database [94] and Matbase (Genomatix) contain well documented, experimentally confirmed promoter modules with synergistic, antagonistic, or additive functions. Comparison with these prior known TF modules can be used to identify and verify meaningful TFBS combinations.

The relative importance of a TFBS is determined by the average ranking order of ECR, prompter and frequency analysis. A TFBS-TG matrix was derived from promoter mining. The score between a TFBS, *Ti *and a gene, *Gj*, is defined as *TFBS *(*Ti*, *Gj*) ∈ < 0,1,2>. 0 means that *Ti *is not present in the promoter of *Gi*; 1 means the presence of a single *Ti *in the conserved promoter regions of *Gi*; 2 means the presence of multiple *Ti *in the conserved promoter regions of *Gi*.

### Knowledge Base and Interaction Search

We collected the positive TF-TG relationships from: Ingenuity knowledge base (Ingenuity), Transfac 11.3 (Biobase) [94], Eldorado (Genomatix), PReMod [95], protein interaction databases HPRD [96] and BioGRID [97]. A TF-TG interaction matrix was formed from the combined resources. Interaction score is defined as *Interaction *(*Ti*, *Gj*) ∈ < 0, 1, 2, 3> The higher the score, the more certainty the TF-TG relationship: 0 means no evidence, 1 indicates the evidence from high throughput screen or computational prediction or gene co-citation from databases ≤10. 2 means supporting evidence is from more than one resources and gene co-citation ≥10. 3 means direct experimental evidence or evidence from multiple resources.

### Data Integration

We calculated the relative confidence score of TF-TG associations by combining the data obtained. One key assumption of our integrative approach is that TGs sharing expression and functional similarity are likely to be regulated by the same TF(s), and TFs sharing expression and functional similarity are likely to form functional modules to regulate the same group of TG(s). We grouped TF using hierarchical clustering, according to an integrated matrix compiled from four types of data sources: a TFBS-TG scoring matrix, a TF-TG functional similarity matrix, a TF-TG expression correlation matrix and a TF-TG interaction matrix. Each value in the four matrices was scaled from 0 to 1 and summed into the integrated TF-TG matrix. The TF-TG matrix was further normalized and scaled between 0 and 1, denoted as *Score *(*Ti*, *Gj*). We grouped TGs into sub-clusters using hierarchical clustering, based on an integrated matrix, combining and capturing information from four data sources: gene expression, TF-TG correlation, promoter TFBS prediction and GO functional similarity. In the integrated matrix, each row represents a gene, and each column represents a feature from one of the four matrices.

We define *Support *between each TF cluster *Ct *and each TG cluster *Cg *as

where *Score*(*Ti*, *Gj*) is from the integrated matrix between TF and TG, *m *is the size of *Ct, n *is the size of *CgTi ∈ Ct*, and *Gj ∈ Cg*.

*Support *describes the connectivity between a TF cluster and a gene cluster. The value of *Support *ranges from 0 to 1. Given a threshold of *Support*, for instance, 0.25, satisfying TF-TG cluster pairs are extracted as correlated cluster pairs. Given a correlated cluster pair, we further define *Confidence *between TF-TG pairs within this cluster pair as

where *L(Ti, Gj) *is calculated by scaling *Score*(*Ti*, *Gj*) into [0.5, 1]. *I(Ti) *is normalized relative TF importance ranging from [0.8, 1.2]. *m *is the size of *Ct, n *is the size of *Cg*, *Ti ∈ Ct *and *Gj ∈ Cg*, and *Ct *and *Cg *are in a cluster pair passed *Support *cutoff. All factors are equally weighted in the equation.

*Confidence *describes the possibility of a true positive TF-TG relationship according to the integrated information. The first factor of *Confidence (Ti, Gj) *denotes the connectivity between a *Ti *from a cluster *Ct *and all genes in a cluster *Cg*, the second factor measures the connectivity between a *Gj *from cluster *Cg *and all TFs in cluster *Ct*, the fourth factor implies the connectivity between *Ti *and *Gj*, and the fifth factor *I(Ti) *denotes the relative importance of *Ti *in our analysis. We rank TFBS-TG pairs based on the normalized *Confidence *score for each TF-TG pair. The TFBS-TG pairs with the highest *Confidence *scores will be selected for experimental validation. For each cluster, we generated a TF-TG association table ranked according to the confidence score. A network graph linking TFs and their TGs was generated using Cytoscape 2.6 http://www.cytoscape.org/.

### Cell Culture, Transfection, and Reporter Gene Assays

The MLE-15 cell is an immortalized mouse lung epithelial cell line that maintains some of the morphological and functional characteristics of type II epithelial cells. MLE-15 cells were cultured in HITES medium [56] for functional characterization of mouse *Elovl1, Slc34a2 *and *Zdhhc3 *promoters. Approximately 1 Kb of the 5'-upstream regulatory regions comprising the proximal promoter were PCR amplified, including the transcription start site (TSS) and a part of the 5'-untranslated (5'-UT) region as depicted in Figure 4. The promoter fragments were confirmed by sequencing from both ends and cloned to generate promoter-luciferase vectors in pGL3-basic plasmid (Promega) and used in transient transfection assays using Fugene 6 at a DNA/Fugene ratio of 1:3 according to the manufacturer's instructions (Roche Applied Science). Briefly, 6-well plates at 30-50% confluence were transfected with a fixed amount of each promoter-luciferase plasmid and various amounts of CMV-based cDNA expressing transactivator plasmids mouse C/EBPα (kind gift from Dr. Mcknight, University of Texas Southwestern Medical Center at Dallas) or human SREBP1c [98]. Total DNA was normalized with corresponding CMV-empty vectors, and transfection efficiency was normalized to β-galactosidase activity using 100 ng/well of pCMV β-galactosidase. Two days after transfection, luciferase and β-galactosidase assays were performed using 20 μl of the supernatant according to a previous protocol [55]. The light units were assayed by luminometry (Berthold Technologies GmbH & Co., Calmbacher, Germany). Data obtained represent the average of three transfection experiments, each carried out in duplicate (*n *= 6) and depicted as means ± S.D. unless stated otherwise.

### Availability

All published microarrays and mouse models we used in this study are listed in Table 1 with references. Unpublished microarray data used in this study are available upon request. Perl scripts for extracting results from Cluster-Buster and confident score calculation can be freely downloaded from http://research.cchmc.org/pbge/jsp/links_v2.jsp

## Abbreviations

ABCA3: ATP-binding cassette sub-family A member 3; ACLY: ATP citrate lyase; ACOXL: acyl-Coenzyme A oxidase-like; ACSS2: acyl-CoA synthetase short-chain family member 2; ADORA2B: adenosine A2b receptor; AHR: aryl hydrocarbon receptor; APP: approximation steps; AQP5: aquaporin 5; BARBIE: barbiturate-inducible element; BCL6B: B-cell CLL/lymphoma 6, member B; BEX2: brain expressed X-linked 2; BIOGRID: Biological General Repository for Interaction Datasets; BTG3: B-cell translocation gene 3; CEBPA: CCAAT/enhancer-binding protein alpha; CFTR: cystic fibrosis transmembrane conductance regulator; CHIP: Chromatin immunoprecipitation; CIZ: Cas-associated zinc finger protein; CLOVER: cis-element over-representation; CMV: Cytomegalovirus; DAVID: Database for Annotation, Visualization and Integrated Discovery; DIRE: Distant Regulatory Elements of co-regulated genes; DLK1: delta-like 1 homolog; ECR: Evolutionarily Conserved Regions; EGF: epidermal growth factor; EGR: Early growth response; ELF5: Ef1alpha-like factor-5; ELOVL1: elongation of very long chain fatty acids-like 1; EMP2: epithelial membrane protein 2; ENAC: epithelial sodium channel; ENPP2: ectonucleotide pyrophosphatase/phosphodiesterase 2; ER: Endoplasmic Reticulum; ERM: ets-related molecule; ERR1: estrogen receptor related 1; ETS: erythroblastosis virus E26 oncogene homolog; ETV5: ETS variant gene 5; FABP5: fatty acid binding protein 5; FLI1: Friend leukemia integration 1; FOXA2: forkhead box A2; GATA6: GATA binding protein 6; GO: Gene Ontology; GPAM: glycerol-3-phosphate acyltransferase, mitochondrial; HES1: hairy and enhancer of split 1; HITES: hydrocortisone, insulin, transferrin, estrogen, and selenium; HNF3: Hepatocyte Nuclear Factor 3; HPRD: Human Protein Reference Database; ID2: inhibitor of DNA binding 2; IRF1: interferon regulatory factor 1; IRFF: Interferon regulatory factors; JUN: v-jun sarcoma virus 17 oncogene homolog; KDR: kinase insert domain protein receptor; KNN: k-Nearest-neighbours; LEF1: lymphoid enhancer binding factor 1; LIPG: lipase, endothelial; LMO2COM: LIM domain only 2 complex; LPCAT1: lysophosphatidylcholine acyltransferase 1; MEF2C: myocyte enhancer factor 2C; MLE-15: Murine lung epithelial cells; MTCH2: mitochondrial carrier homolog 2; NF1: nuclear factor I; NFAT: Nuclear factor of activated T-cells; NFATC3: nuclear factor of activated T-cells, calcineurin-dependent 3; NFE2: nuclear factor, erythroid derived 2; NFKB: nuclear factor of kappa light polypeptide gene enhancer in B-cells; NKX2-1: NK2 homeobox 1; NLP: Natural Language Processing; NOTCH1: neurogenic locus notch homolog protein 1; NPT2B: Na(+)/Pi co-transporter 2B; NR1H2/3: nuclear receptor subfamily 1, group H, member 2/3; OCT1: organic cation transporter 1; PDIA5: protein disulfide isomerase associated 5; POU2F1: POU domain, class 2, transcription factor 1; PPAR: peroxisome proliferator-activated receptor; PRDX6: peroxiredoxin 6; PREMOD: predicted transcriptional regulatory modules; QT: Quality Threshold; RAR: retinoic acid receptor; RUNX1T1: runt-related transcription factor 1; translocated to, 1; RVISTA: Rank Vista; S.D.: Standard Deviation; SCAP: SREBP cleavage-activating protein; SCD: stearoyl-Coenzyme A desaturase; SCGB1A1: secretoglobin, family 1A, member 1; SLC34A2: solute carrier family 34 (sodium phosphate), member 2; SOAT1: sterol O-acyltransferase 1; SOX9: Sex determining region Y-Box 9; SP1: Sp1 transcription factor (specificity protein 1); SPP1: secreted phosphoprotein 1; SREBP: Sterol Regulatory Element Binding Proteins; SREPINB9: serpin peptidase inhibitor, clade B (ovalbumin), member 9; STAT6: signal transducer and activator of transcription 6; STFPA: surfactant, pulmonary-associated protein A; STFPB: surfactant, pulmonary-associated protein B; STFPC: surfactant, pulmonary-associated protein C; STFPD: surfactant, pulmonary-associated protein D; TCF4: transcription factor 4; TF: Transcription Factor; TFBS: Transcription Factor Binding Site; TG: Target Gene; TRANSFAC: Transcriptional Factor Database; TSS: Transcription start site; TTF-1: thyroid transcription factor 1; VEGFA: vascular endothelial growth factor A; WARS: tryptophanyl-tRNA synthetase; WWTR1: WW domain containing transcription regulator 1; ZDHHC3: zinc finger, DHHC domain containing 3.

## Authors' contributions

YX designed and coordinated the overall project, participated in the statistical analysis and drafted the manuscript. MZ and LJL participated in the design; drafting and computational analysis of the data integration section. YW carried out multiple data analysis and assisted manuscript preparation. PK assisted the data analysis and manuscript preparation. VD carried out promoter reporter assays and wrote that part of the manuscript. JAW provided mRNA data, contributed to study design and to the writing and revising of the manuscript. All authors read and approved the final manuscript.

## Supplementary Material

Additional file 1 **Data collecting and Clustering**. Click here for file

Additional file 2 **Support & Confidence Calculation For C1 Genes**. Click here for file

Additional file 3 **Support & Confidence Calculation For C2 Genes**. Click here for file

Additional file 4 **Support & Confidence Calculation For C28 Genes**. Click here for file

Additional file 5 **Top Ranked CEBP Targets According To The Integrative Score**. Click here for file

Additional file 6 **Top Ranked SREBP Targets According To The Integrative Score**. Click here for file

Additional file 7 **Top Ranked HNF3 Targets According To The Integrative Score**. Click here for file
