# Supplementary material for: A systems approach to mapping transcriptional networks controlling surfactant homeostasis
Source: BMC Genomics. 2010 Jul 26;11:451. doi: 10.1186/1471-2164-11-451 (PMC3091648; doi:10.1186/1471-2164-11-451)
Supplement: Additional file 5 — Top Ranked CEBP Targets According To The Integrative Score. [file 1471-2164-11-451-S5.DOC]

**Additional file 5 - Top Ranked CEBP Targets According To The Integrative Score**

| **Top 100 CEBP Candidate Targets** | | | | | **Top 100 CEBP Candidate Targets (Unknown)** | | | |
| --- | --- | --- | --- | --- | --- | --- | --- | --- |
| **Gj** | **Score** | **Cluster** | **Cebpa Array** | **Reference** | **Gj** | **Score** | **Cluster** | **Cebpa Array** |
| CEBPA | 0.82 | C1C2C28 | -6.6 | Kyrmizi et al. 2006 | CDKN2B | 0.73 | C28 | -2.1 |
| FOXA2 | 0.77 | C1C2 |  | Martis et al 2006 | EXOSC7 | 0.72 | C2 |  |
| FOS | 0.76 | C28 | -1.3 | Cammenga et al. 2003 | DLK1 | 0.72 | C2C28 | -3.4 |
| SREBF1 | 0.74 | C1C2C28 | -1.3 | Le Lay et al. 2002 | AHR | 0.71 | C1C28 |  |
| STAT3 | 0.73 | C28 |  | Numata et al. 2005 | SOX2 | 0.70 | C28 |  |
| JUN | 0.73 | C1C28 |  | Rangatia et al. 2002 | KIT | 0.70 | C2C28 |  |
| CDKN2B | 0.73 | C28 | -2.1 |  | BEX2 | 0.69 | C2C28 | -9.4 |
| EXOSC7 | 0.72 | C2 |  |  | CYP2E1 | 0.69 | C1C2C28 | -1.5 |
| DLK1 | 0.72 | C2C28 | -3.4 |  | ETS1 | 0.69 | C1C28 |  |
| AHR | 0.71 | C1C28 |  |  | KLF7 | 0.69 | C1 |  |
| SFTPB | 0.71 | C2C28 | -2.2 | Martis et al. 2006 | WWTR1 | 0.68 | C1C28 |  |
| HCK | 0.71 | C2C28 | -1.5 | Komuro et al. 2003 | CES3 | 0.68 | C28 |  |
| VLDLR | 0.70 | C1C28 |  | Kreuter et al. 1999 | PRDM1 | 0.68 | C1C28 |  |
| SOX2 | 0.70 | C28 |  |  | MEF2C | 0.68 | C1 |  |
| KIT | 0.70 | C2C28 |  |  | SFTPC | 0.68 | C1 |  |
| SPP1 | 0.70 | C28 |  | Liu et al. 2004 | FOXA1 | 0.68 | C1 | 1.5 |
| MYB | 0.69 | C28 |  | Verbeek, et al. 1999 | ACTB | 0.68 | C1 |  |
| BEX2 | 0.69 | C2C28 | -9.4 |  | FOXO3 | 0.67 | C28 |  |
| VEGFA | 0.69 | C1C28 | -1.3 | Nolan et al. 2004 | TIMP3 | 0.66 | C1 | -1.5 |
| SFTPA1 | 0.69 | C1C2 | -17.3 | Martis et al. 2006 | PVRL3 | 0.66 | C1 |  |
| ID2 | 0.69 | C1 |  | Tavor et al. 2003 | PCX | 0.66 | C1C28 |  |
| CYP2E1 | 0.69 | C1C2C28 | -1.5 |  | FGFR2 | 0.66 | C1C28 | -1.8 |
| ETS1 | 0.69 | C1C28 |  |  | FABP5 | 0.66 | C2C28 | -2.5 |
| EBF1 | 0.69 | C1 | -1.2 | Jimenez, et al. 2007 | TCFCP2L1 | 0.65 | C28 | -2.1 |
| KLF7 | 0.69 | C1 |  |  | FLI1 | 0.65 | C1 |  |
| WWTR1 | 0.68 | C1C28 |  |  | IER3 | 0.65 | C28 | -1.6 |
| CES3 | 0.68 | C28 |  |  | C6 | 0.65 | C1 |  |
| PRDM1 | 0.68 | C1C28 |  |  | HC | 0.65 | C2C28 | -8.1 |
| MEF2C | 0.68 | C1 |  |  | BDNF | 0.65 | C1 |  |
| SFTPC | 0.68 | C1 |  |  | 1190002N15RIK | 0.64 | C1 |  |
| FOXA1 | 0.68 | C1 | 1.5 |  | SMAD5 | 0.64 | C1 |  |
| ACTB | 0.68 | C1 |  |  | TMOD3 | 0.64 | C1 |  |
| RUNX1T1 | 0.68 | C1 |  | Rochford et al 2004 | CBFA2T3 | 0.64 | C28 |  |
| MAPK14 | 0.67 | C28 |  | Kumar et al. 2003 | ME1 | 0.64 | C28 | -1.5 |
| FOXO3 | 0.67 | C28 |  |  | ANKIB1 | 0.64 | C28 |  |
| TIMP3 | 0.66 | C1 | -1.5 |  | KLF9 | 0.64 | C28 |  |
| PVRL3 | 0.66 | C1 |  |  | RAMP2 | 0.64 | C1 |  |
| PCX | 0.66 | C1C28 |  |  | ABCC3 | 0.64 | C28 |  |
| FGFR2 | 0.66 | C1C28 | -1.8 |  | ACOXL | 0.63 | C1C2 | -6.0 |
| FABP5 | 0.66 | C2C28 | -2.5 |  | SIVA1 | 0.63 | C1C28 |  |
| TCFCP2L1 | 0.65 | C28 | -2.1 |  | FAH | 0.63 | C28 |  |
| FLI1 | 0.65 | C1 |  |  | LPCAT1 | 0.63 | C2C28 |  |
| CFTR | 0.65 | C1 | -1.7 | Pittman et al,1995 | SERPINF1 | 0.63 | C2C28 | -2.0 |
| FASN | 0.65 | C28 | -1.5 | Martis et al 2006 | LRP2 | 0.63 | C1C2C28 | -2.2 |
| IER3 | 0.65 | C28 | -1.6 |  | ELF5 | 0.63 | C1 | -1.5 |
| C6 | 0.65 | C1 |  |  | ABCB1A | 0.63 | C1 |  |
| HC | 0.65 | C2C28 | -8.1 |  | GNA14 | 0.62 | C28 |  |
| BDNF | 0.65 | C1 |  |  | LIPA | 0.62 | C1C28 |  |
| 1190002N15RIK | 0.64 | C1 |  |  | QK | 0.62 | C1C28 |  |
| SMAD5 | 0.64 | C1 |  |  | B3GNT2 | 0.62 | C1C28 |  |
| FOXF1A | 0.64 | C1 |  | Kim et al, 2005 | ETV5 | 0.62 | C1 |  |
| TMOD3 | 0.64 | C1 |  |  | MBP | 0.62 | C28 |  |
| CBFA2T3 | 0.64 | C28 |  |  | MEG3 | 0.62 | C1C28 | -1.5 |
| ME1 | 0.64 | C28 | -1.5 |  | MSN | 0.62 | C1 |  |
| ANKIB1 | 0.64 | C28 |  |  | HMGCS1 | 0.62 | C1 |  |
| KLF9 | 0.64 | C28 |  |  | YWHAB | 0.62 | C1 |  |
| SCD1 | 0.64 | C2 | -18.7 | Christy et al. 1989 | MMD | 0.62 | C1 |  |
| RAMP2 | 0.64 | C1 |  |  | GADD45G | 0.62 | C28 |  |
| ABCC3 | 0.64 | C28 |  |  | S100G | 0.61 | C2C28 | -17.8 |
| ACOXL | 0.63 | C1C2 | -6.0 |  | SUPT16H | 0.61 | C1 |  |
| SIVA1 | 0.63 | C1C28 |  |  | 3110001I20RIK | 0.61 | C1 | -1.5 |
| FAH | 0.63 | C28 |  |  | ZNRF2 | 0.61 | C1C28 |  |
| LPCAT1 | 0.63 | C2C28 |  |  | RTN1 | 0.61 | C1C28 |  |
| SERPINF1 | 0.63 | C2C28 | -2.0 |  | RCAN1 | 0.61 | C1C2C28 |  |
| LRP2 | 0.63 | C1C2C28 | -2.2 |  | VSNL1 | 0.61 | C1C28 |  |
| ELF5 | 0.63 | C1 | -1.5 |  | SCIN | 0.61 | C1 |  |
| ABCB1A | 0.63 | C1 |  |  | SLC34A2 | 0.61 | C2C28 | -7.5 |
| GNA14 | 0.62 | C28 |  |  | TACSTD2 | 0.61 | C28 | -1.6 |
| LIPA | 0.62 | C1C28 |  |  | PRDX6 | 0.61 | C1C2 |  |
| CST8 | 0.62 | C1 |  | Hsia and Cornwall 2001 | CAR8 | 0.61 | C2C28 | -2.9 |
| QK | 0.62 | C1C28 |  |  | SERPINB6B | 0.61 | C1 |  |
| B3GNT2 | 0.62 | C1C28 |  |  | SERPINB9 | 0.61 | C1C2C28 |  |
| ETV5 | 0.62 | C1 |  |  | RAB2 | 0.61 | C1 |  |
| MBP | 0.62 | C28 |  |  | ELOVL1 | 0.60 | C1C2 | -1.4 |
| MEG3 | 0.62 | C1C28 | -1.5 |  | ITM2A | 0.60 | C28 |  |
| MSN | 0.62 | C1 |  |  | TGM2 | 0.60 | C1C28 |  |
| HMGCS1 | 0.62 | C1 |  |  | SLK | 0.60 | C1 |  |
| YWHAB | 0.62 | C1 |  |  | GOLPH2 | 0.60 | C1 |  |
| MMD | 0.62 | C1 |  |  | NGDN | 0.60 | C1 |  |
| GADD45G | 0.62 | C28 |  |  | CD38 | 0.60 | C1C2C28 |  |
| SCGB1A1 | 0.62 | C28 |  | Cassel et al 2000 | ACSL4 | 0.60 | C1 | -1.5 |
| S100G | 0.61 | C2C28 | -17.8 |  | EMP2 | 0.60 | C2C28 | -1.5 |
| CD36 | 0.61 | C28 | -1.5 | Qiao et al. 2008 | GPAM | 0.60 | C1C2C28 | -1.9 |
| SUPT16H | 0.61 | C1 |  |  | TSPAN2 | 0.60 | C1 |  |
| 3110001I20RIK | 0.61 | C1 | -1.5 |  | A130022J15RIK | 0.60 | C1 |  |
| ZNRF2 | 0.61 | C1C28 |  |  | 1190002H23RIK | 0.60 | C1C28 | -2.0 |
| RTN1 | 0.61 | C1C28 |  |  | CPD | 0.59 | C1 |  |
| RCAN1 | 0.61 | C1C2C28 |  |  | RTKN2 | 0.59 | C1C2 | -3.4 |
| VSNL1 | 0.61 | C1C28 |  |  | AQP5 | 0.59 | C1C2 | -7.0 |
| SCIN | 0.61 | C1 |  |  | PODXL | 0.59 | C1 |  |
| SLC34A2 | 0.61 | C2C28 | -7.5 |  | CCNG2 | 0.59 | C1 |  |
| TACSTD2 | 0.61 | C28 | -1.6 |  | RGS2 | 0.59 | C1 |  |
| PRDX6 | 0.61 | C1C2 |  |  | FDPS | 0.59 | C28 | -1.5 |
| CAR8 | 0.61 | C2C28 | -2.9 |  | SCHIP1 | 0.59 | C1C28 |  |
| SERPINB6B | 0.61 | C1 |  |  | SYTL2 | 0.59 | C1 | -1.6 |
| SERPINB9 | 0.61 | C1C2C28 |  |  | SCD2 | 0.59 | C1C28 | -1.9 |
| LCN2 | 0.61 | C28 |  | Numata et al. 2005 | GNG2 | 0.59 | C1 |  |
| RAB2 | 0.61 | C1 |  |  | NDST1 | 0.59 | C1C28 |  |
| ELOVL1 | 0.60 | C1C2 | -1.4 |  | SCNN1B | 0.59 | C1 | -1.4 |
| ITM2A | 0.60 | C28 |  |  | PION | 0.59 | C1 | -1.7 |
