# Supplementary material for: A systems approach to mapping transcriptional networks controlling surfactant homeostasis
Source: BMC Genomics. 2010 Jul 26;11:451. doi: 10.1186/1471-2164-11-451 (PMC3091648; doi:10.1186/1471-2164-11-451)
Supplement: Additional file 6 — Top Ranked SREBP Targets According To The Integrative Score. [file 1471-2164-11-451-S6.DOC]

**Additional file 6 - Top Ranked SREBP Targets According To The Integrative Score**

| **Top 100 SREBP Candidate Targets** | | | |  | **Top 100 SREBP Candidate Targets (Unknown)** | | | |
| --- | --- | --- | --- | --- | --- | --- | --- | --- |
| **Gj** | **Score** | **Cluster** | **Scap Array** | **Reference** | **Gj** | **Score** | **Cluster** | **Scap Array** |
| FASN | 0.73 | C28 | -1.6 | Sun et al. 2002 | FDPS | 0.69 | C28 | -1.6 |
| GPAM | 0.72 | C1C2C28 | -1.4 | Ericsson et al. 1997 | FOXA2 | 0.68 | C1C2 |  |
| SOAT1 | 0.72 | C1C2C28 | -1.3 | Farrell, 2005 | LPHN3 | 0.67 | C1 |  |
| SCD1 | 0.72 | C2 | -6.1 | Horton et al. 2002 | STARD4 | 0.67 | C1 | -1.3 |
| SCD2 | 0.71 | C1C2C28 | -1.8 | Tabor et al. 1999 | S100G | 0.66 | C2C28 |  |
| SREBF1 | 0.71 | C1C2C28 | -1.6 | She 2005 | DLK1 | 0.66 | C2C28 | -1.7 |
| FDPS | 0.69 | C28 | -1.6 |  | JUN | 0.66 | C1C28 |  |
| FOXA2 | 0.68 | C1C2 |  |  | STAT3 | 0.66 | C28 |  |
| HDC | 0.68 | C28 | -1.4 | Ai et al. 2006 | SCNN1B | 0.66 | C1 |  |
| AHR | 0.67 | C1C28 |  | Iwano et al. 2005 | MTCH2 | 0.65 | C1C2C28 |  |
| LPHN3 | 0.67 | C1 |  |  | 2310046K01RIK | 0.65 | C1 |  |
| STARD4 | 0.67 | C1 | -1.3 |  | ELOVL1 | 0.65 | C1C2 |  |
| S100G | 0.66 | C2C28 |  |  | ENPP2 | 0.65 | C1C28 | -1.4 |
| DLK1 | 0.66 | C2C28 | -1.7 |  | CDKN2B | 0.64 | C28 |  |
| JUN | 0.66 | C1C28 |  |  | FABP5 | 0.64 | C2C28 | -1.9 |
| STAT3 | 0.66 | C28 |  |  | CST8 | 0.64 | C1 | -2.1 |
| CEBPA | 0.66 | C1C2C28 |  | Pedersen et al. 2007 | MEF2C | 0.64 | C1 |  |
| SCNN1B | 0.66 | C1 |  |  | BCL6B | 0.63 | C1 |  |
| MTCH2 | 0.65 | C1C2C28 |  |  | WWTR1 | 0.63 | C1C28 |  |
| ID2 | 0.65 | C1 | -1.4 | Moldes et al. 1999 | 6330416G13RIK | 0.63 | C1C28 |  |
| 2310046K01RIK | 0.65 | C1 |  |  | KLF7 | 0.62 | C1 |  |
| ELOVL1 | 0.65 | C1C2 |  |  | ABCA3 | 0.62 | C1C2 | -1.6 |
| ENPP2 | 0.65 | C1C28 | -1.4 |  | SERPINB9 | 0.62 | C1C2C28 |  |
| CDKN2B | 0.64 | C28 |  |  | KLF9 | 0.62 | C28 |  |
| FABP5 | 0.64 | C2C28 | -1.9 |  | MID1IP1 | 0.62 | C2 | -1.5 |
| CST8 | 0.64 | C1 | -2.1 |  | ETV5 | 0.62 | C1 |  |
| MEF2C | 0.64 | C1 |  |  | CLDN18 | 0.62 | C1 |  |
| BCL6B | 0.63 | C1 |  |  | ZDHHC3 | 0.62 | C1C2 |  |
| WWTR1 | 0.63 | C1C28 |  |  | SOX2 | 0.61 | C28 |  |
| 6330416G13RIK | 0.63 | C1C28 |  |  | SIVA1 | 0.61 | C1C28 |  |
| KLF7 | 0.62 | C1 |  |  | FOS | 0.61 | C28 |  |
| ABCA3 | 0.62 | C1C2 | -1.6 |  | SFTPB | 0.61 | C2C28 | -1.3 |
| SERPINB9 | 0.62 | C1C2C28 |  |  | DTNA | 0.61 | C1C28 |  |
| KLF9 | 0.62 | C28 |  |  | 1200002N14RIK | 0.61 | C1 |  |
| MID1IP1 | 0.62 | C2 | -1.5 |  | S100A14 | 0.61 | C1 |  |
| ETV5 | 0.62 | C1 |  |  | EMP2 | 0.61 | C2C28 |  |
| CLDN18 | 0.62 | C1 |  |  | PRDX6 | 0.61 | C1C2 |  |
| ZDHHC3 | 0.62 | C1C2 |  |  | LIPG | 0.61 | C1C28 | -1.5 |
| SOX2 | 0.61 | C28 |  |  | ICAM2 | 0.61 | C1 |  |
| SIVA1 | 0.61 | C1C28 |  |  | 1110032E23RIK | 0.61 | C1 |  |
| FOS | 0.61 | C28 |  |  | ACTB | 0.61 | C1 |  |
| SFTPB | 0.61 | C2C28 | -1.3 |  | SYTL2 | 0.61 | C1 |  |
| DTNA | 0.61 | C1C28 |  |  | EXOSC7 | 0.60 | C2 |  |
| 1200002N14RIK | 0.61 | C1 |  |  | FAH | 0.60 | C28 |  |
| S100A14 | 0.61 | C1 |  |  | NTN1 | 0.60 | C1 |  |
| EMP2 | 0.61 | C2C28 |  |  | CYP4V3 | 0.60 | C2C28 | -1.5 |
| PRDX6 | 0.61 | C1C2 |  |  | FOXO3 | 0.60 | C28 |  |
| LIPG | 0.61 | C1C28 | -1.5 |  | SDC2 | 0.60 | C1 |  |
| ICAM2 | 0.61 | C1 |  |  | MYB | 0.60 | C28 |  |
| 1110032E23RIK | 0.61 | C1 |  |  | AQP1 | 0.60 | C28 |  |
| ACTB | 0.61 | C1 |  |  | LPCAT1 | 0.60 | C2C28 | -1.5 |
| ADH1 | 0.61 | C1 |  | He et al. 2004 | PARD6B | 0.60 | C1 |  |
| SYTL2 | 0.61 | C1 |  |  | ETS1 | 0.60 | C1C28 |  |
| EXOSC7 | 0.60 | C2 |  |  | TBX4 | 0.60 | C1 |  |
| FAH | 0.60 | C28 |  |  | SERPINF1 | 0.60 | C2C28 |  |
| NTN1 | 0.60 | C1 |  |  | CBFA2T3 | 0.60 | C28 |  |
| CYP4V3 | 0.60 | C2C28 | -1.5 |  | TCFCP2L1 | 0.60 | C28 |  |
| VEGFA | 0.60 | C1C28 |  | Sun et al. 2002 | CD200 | 0.59 | C28 |  |
| FOXO3 | 0.60 | C28 |  |  | BDNF | 0.59 | C1 |  |
| SDC2 | 0.60 | C1 |  |  | SEMA7A | 0.59 | C1 |  |
| MYB | 0.60 | C28 |  |  | ROS1 | 0.59 | C1 | -1.8 |
| AQP1 | 0.60 | C28 |  |  | ACOXL | 0.59 | C1C2 | -4.3 |
| LPCAT1 | 0.60 | C2C28 | -1.5 |  | ZDHHC14 | 0.59 | C1 |  |
| PARD6B | 0.60 | C1 |  |  | NGDN | 0.59 | C1 |  |
| ETS1 | 0.60 | C1C28 |  |  | CTGF | 0.59 | C1C28 |  |
| TBX4 | 0.60 | C1 |  |  | TGOLN1 | 0.59 | C1C28 |  |
| SERPINF1 | 0.60 | C2C28 |  |  | PRDM1 | 0.59 | C1C28 |  |
| CBFA2T3 | 0.60 | C28 |  |  | BEX2 | 0.59 | C2C28 | -1.7 |
| TCFCP2L1 | 0.60 | C28 |  |  | HMGCS1 | 0.59 | C1 | -1.6 |
| CD200 | 0.59 | C28 |  |  | NDST1 | 0.59 | C1C28 |  |
| BDNF | 0.59 | C1 |  |  | AQP5 | 0.59 | C1C2 |  |
| SEMA7A | 0.59 | C1 |  |  | SOX7 | 0.59 | C1 |  |
| ROS1 | 0.59 | C1 | -1.8 |  | GDPD2 | 0.58 | C1C2 | -1.9 |
| ACOXL | 0.59 | C1C2 | -4.3 |  | SMAD5 | 0.58 | C1 |  |
| ZDHHC14 | 0.59 | C1 |  |  | UPK3B | 0.58 | C1 |  |
| NGDN | 0.59 | C1 |  |  | AGER | 0.58 | C28 |  |
| CTGF | 0.59 | C1C28 |  |  | RNASE4 | 0.58 | C2C28 |  |
| TGOLN1 | 0.59 | C1C28 |  |  | CENTD3 | 0.58 | C1 |  |
| PRDM1 | 0.59 | C1C28 |  |  | ARRB1 | 0.58 | C1 |  |
| BEX2 | 0.59 | C2C28 | -1.7 |  | COL4A4 | 0.58 | C1C28 |  |
| HMGCS1 | 0.59 | C1 | -1.6 |  | CDH5 | 0.58 | C1 |  |
| NDST1 | 0.59 | C1C28 |  |  | IER3 | 0.58 | C28 |  |
| AQP5 | 0.59 | C1C2 |  |  | KLRA3 | 0.58 | C1 |  |
| SOX7 | 0.59 | C1 |  |  | FAM13C | 0.58 | C1 |  |
| GDPD2 | 0.58 | C1C2 | -1.9 |  | MYCT1 | 0.57 | C1 |  |
| SMAD5 | 0.58 | C1 |  |  | COL6A1 | 0.57 | C1 |  |
| UPK3B | 0.58 | C1 |  |  | FLI1 | 0.57 | C1 |  |
| AGER | 0.58 | C28 |  |  | SLC34A2 | 0.57 | C2C28 |  |
| RNASE4 | 0.58 | C2C28 |  |  | NPC1 | 0.57 | C1C28 | -1.3 |
| CENTD3 | 0.58 | C1 |  |  | RUNX1T1 | 0.57 | C1 |  |
| ARRB1 | 0.58 | C1 |  |  | CXCR4 | 0.57 | C1 |  |
| COL4A4 | 0.58 | C1C28 |  |  | RCAN1 | 0.57 | C1C2 |  |
| CDH5 | 0.58 | C1 |  |  | SERPINB6B | 0.57 | C1 |  |
| IER3 | 0.58 | C28 |  |  | LIPA | 0.57 | C1C28 |  |
| KLRA3 | 0.58 | C1 |  |  | SUPT16H | 0.57 | C1 |  |
| FAM13C | 0.58 | C1 |  |  | ITGA3 | 0.57 | C28 |  |
| MYCT1 | 0.57 | C1 |  |  | CFTR | 0.57 | C1 |  |
| COL6A1 | 0.57 | C1 |  |  | LGALS9 | 0.57 | C28 |  |
| FLI1 | 0.57 | C1 |  |  | HBA-A1 | 0.57 | C1 |  |
| SLC34A2 | 0.57 | C2C28 |  |  | CYP2E1 | 0.57 | C1 |  |
