# Supplementary material for: A systems approach to mapping transcriptional networks controlling surfactant homeostasis
Source: BMC Genomics. 2010 Jul 26;11:451. doi: 10.1186/1471-2164-11-451 (PMC3091648; doi:10.1186/1471-2164-11-451)
Supplement: Additional file 7 — Top Ranked HNF3 Targets According To The Integrative Score. [file 1471-2164-11-451-S7.DOC]

**Additional file 7 -** Top Ranked HNF3 Targets According To The Integrative Score

| **Top 100 Foxa1/2 Candidate Targets** | | | |  | **Top 100 Foxa1/2 Candidates (Unknown)** | | | |
| --- | --- | --- | --- | --- | --- | --- | --- | --- |
| **Gj** | **Score** | **Cluster** | **Foxa2 Array** | **Reference** | **Gj** | **Score** | **Cluster** | **Foxa2 Array** |
| FOXA2 | 0.73 | C1C2 | -10.9 | Rausa et al. 2000 | SFTPA1 | 0.70 | C1C2 |  |
| SFTPB | 0.73 | C2C28 | -1.6 | Clevidence et al. 1994 | ACTB | 0.68 | C1 |  |
| CEBPA | 0.72 | C1C2C28 |  | Martis et al. 2006 | BDNF | 0.68 | C1 |  |
| SFTPA1 | 0.70 | C1C2 |  |  | CCKAR | 0.68 | C1 | -1.8 |
| SFTPC | 0.69 | C1 | 1.5 | Minoo et al. 2007 | SREBF1 | 0.67 | C1C2C28 |  |
| FOXA1 | 0.68 | C1 | 1.9 | Rausa et al. 2000 | ID4 | 0.67 | C1 |  |
| ACTB | 0.68 | C1 |  |  | CXCR4 | 0.66 | C1 |  |
| BDNF | 0.68 | C1 |  |  | AZIN1 | 0.64 | C1C28 |  |
| DLK1 | 0.68 | C2C28 | -2.9 | Wolfrum et al. 2003 | HMGCS1 | 0.64 | C1 | -1.7 |
| CCKAR | 0.68 | C1 | -1.8 |  | BTBD3 | 0.64 | C1 |  |
| SREBF1 | 0.67 | C1C2C28 |  |  | ACTG2 | 0.64 | C1 |  |
| ID4 | 0.67 | C1 |  |  | RCAN1 | 0.63 | C1C2C28 |  |
| CXCR4 | 0.66 | C1 |  |  | MNS1 | 0.63 | C1C28 |  |
| KITL | 0.65 | C1 |  | Rao et al. 1996 | SOX2 | 0.63 | C28 |  |
| AZIN1 | 0.64 | C1C28 |  |  | MAPK14 | 0.63 | C28 |  |
| HMGCS1 | 0.64 | C1 | -1.7 |  | BEX2 | 0.63 | C2 | -15.7 |
| BTBD3 | 0.64 | C1 |  |  | HC | 0.63 | C2 | -11.3 |
| ACTG2 | 0.64 | C1 |  |  | NTN1 | 0.62 | C1 | -1.5 |
| RCAN1 | 0.63 | C1C2C28 |  |  | MACF1 | 0.62 | C1 |  |
| MNS1 | 0.63 | C1C28 |  |  | CXCL15 | 0.62 | C1 |  |
| SOX2 | 0.63 | C28 |  |  | SEMA6D | 0.62 | C1 |  |
| MAPK14 | 0.63 | C28 |  |  | GNG2 | 0.62 | C1 |  |
| BEX2 | 0.63 | C2 | -15.7 |  | KLF7 | 0.62 | C1 |  |
| HC | 0.63 | C2 | -11.3 |  | JUN | 0.62 | C1C28 |  |
| NTN1 | 0.62 | C1 | -1.5 |  | FOXO3 | 0.62 | C28 |  |
| MACF1 | 0.62 | C1 |  |  | WWTR1 | 0.62 | C1C28 |  |
| CXCL15 | 0.62 | C1 |  |  | ANXA6 | 0.61 | C1 |  |
| SEMA6D | 0.62 | C1 |  |  | ETV5 | 0.61 | C1 |  |
| GNG2 | 0.62 | C1 |  |  | PODXL | 0.61 | C1 |  |
| KLF7 | 0.62 | C1 |  |  | DOCK9 | 0.61 | C1C28 |  |
| JUN | 0.62 | C1C28 |  |  | DTNA | 0.61 | C1C28 |  |
| FOXO3 | 0.62 | C28 |  |  | CDKN2B | 0.61 | C28 |  |
| WWTR1 | 0.62 | C1C28 |  |  | C6 | 0.61 | C1 |  |
| ANXA6 | 0.61 | C1 |  |  | CYP2E1 | 0.61 | C1C2 | -1.6 |
| ETV5 | 0.61 | C1 |  |  | CLDN18 | 0.61 | C1 | -1.6 |
| PODXL | 0.61 | C1 |  |  | ANXA3 | 0.61 | C1 |  |
| FOXF1A | 0.61 | C1 |  | Kim et al. 2005 | GNAI1 | 0.61 | C1 |  |
| DOCK9 | 0.61 | C1C28 |  |  | EDIL3 | 0.61 | C1 | -1.6 |
| DTNA | 0.61 | C1C28 |  |  | ABCC3 | 0.60 | C28 | 1.6 |
| CDKN2B | 0.61 | C28 |  |  | MTDH | 0.60 | C1 |  |
| C6 | 0.61 | C1 |  |  | TCFCP2L1 | 0.60 | C28 |  |
| CYP2E1 | 0.61 | C1C2 | -1.6 |  | LIPA | 0.60 | C1C28 |  |
| CLDN18 | 0.61 | C1 | -1.6 |  | MRVI1 | 0.60 | C1 |  |
| ANXA3 | 0.61 | C1 |  |  | EXOSC7 | 0.60 | C2 |  |
| GNAI1 | 0.61 | C1 |  |  | FOS | 0.60 | C28 |  |
| EDIL3 | 0.61 | C1 | -1.6 |  | RUNX1T1 | 0.60 | C1 |  |
| ABCC3 | 0.60 | C28 | 1.6 |  | 3110001I20RIK | 0.60 | C1 |  |
| MTDH | 0.60 | C1 |  |  | CD34 | 0.60 | C1 |  |
| TCFCP2L1 | 0.60 | C28 |  |  | GGCX | 0.60 | C1C2C28 |  |
| LIPA | 0.60 | C1C28 |  |  | EHD2 | 0.60 | C1 |  |
| MRVI1 | 0.60 | C1 |  |  | QK | 0.60 | C1C28 |  |
| EXOSC7 | 0.60 | C2 |  |  | DDX39 | 0.60 | C1 |  |
| FOS | 0.60 | C28 |  |  | PDIA5 | 0.60 | C1C2C28 |  |
| RUNX1T1 | 0.60 | C1 |  |  | FHL1 | 0.60 | C1C28 |  |
| 3110001I20RIK | 0.60 | C1 |  |  | GNB4 | 0.60 | C1 |  |
| CD34 | 0.60 | C1 |  |  | A830073O21RIK | 0.59 | C1 |  |
| GGCX | 0.60 | C1C2C28 |  |  | STAT3 | 0.59 | C28 |  |
| EHD2 | 0.60 | C1 |  |  | PRDM1 | 0.59 | C1C28 |  |
| QK | 0.60 | C1C28 |  |  | BTG3 | 0.59 | C1C2 |  |
| DDX39 | 0.60 | C1 |  |  | DNAHC8 | 0.59 | C1 | -1.6 |
| ABCA3 | 0.60 | C1C2 | -1.8 | Besnard et al. 2007 | GABARAPL1 | 0.59 | C1C28 |  |
| PDIA5 | 0.60 | C1C2C28 |  |  | ACTA2 | 0.59 | C1 |  |
| FHL1 | 0.60 | C1C28 |  |  | SMAD5 | 0.59 | C1 |  |
| GNB4 | 0.60 | C1 |  |  | ZFX | 0.59 | C1 |  |
| CFTR | 0.60 | C1 |  | Levinson et al. 1997 | ETS1 | 0.59 | C1C28 |  |
| A830073O21RIK | 0.59 | C1 |  |  | HBA-A1 | 0.59 | C1 | -4.8 |
| STAT3 | 0.59 | C28 |  |  | COL6A1 | 0.59 | C1 |  |
| PRDM1 | 0.59 | C1C28 |  |  | F2R | 0.59 | C1 |  |
| BTG3 | 0.59 | C1C2 |  |  | CLDN10 | 0.59 | C1 |  |
| DNAHC8 | 0.59 | C1 | -1.6 |  | ELOVL1 | 0.59 | C1C2 |  |
| GABARAPL1 | 0.59 | C1C28 |  |  | CNN3 | 0.59 | C1 |  |
| ACTA2 | 0.59 | C1 |  |  | SUPT16H | 0.59 | C1 |  |
| SMAD5 | 0.59 | C1 |  |  | FLI1 | 0.58 | C1 |  |
| ZFX | 0.59 | C1 |  |  | KDR | 0.58 | C1C2C28 |  |
| ETS1 | 0.59 | C1C28 |  |  | MAPRE1 | 0.58 | C1 |  |
| HBA-A1 | 0.59 | C1 | -4.8 |  | YWHAB | 0.58 | C1 |  |
| COL6A1 | 0.59 | C1 |  |  | MGLL | 0.58 | C1 |  |
| F2R | 0.59 | C1 |  |  | ZNRF2 | 0.58 | C1C28 |  |
| CLDN10 | 0.59 | C1 |  |  | KLF9 | 0.58 | C28 |  |
| ELOVL1 | 0.59 | C1C2 |  |  | 2810003C17RIK | 0.58 | C1C2 |  |
| CNN3 | 0.59 | C1 |  |  | CLMN | 0.58 | C1 | -1.8 |
| SUPT16H | 0.59 | C1 |  |  | SLC23A2 | 0.58 | C1C28 |  |
| FLI1 | 0.58 | C1 |  |  | SH3D19 | 0.58 | C1C28 |  |
| KDR | 0.58 | C1C2C28 |  |  | UPK3B | 0.58 | C1 |  |
| MAPRE1 | 0.58 | C1 |  |  | PCOLCE2 | 0.58 | C1 |  |
| YWHAB | 0.58 | C1 |  |  | PALM2 | 0.58 | C1C2 | -1.9 |
| MGLL | 0.58 | C1 |  |  | TSN | 0.58 | C1 |  |
| ZNRF2 | 0.58 | C1C28 |  |  | JAM2 | 0.58 | C1 |  |
| PCX | 0.58 | C1C28 | 1.6 | Boonsaen et al. 2007 | FBLIM1 | 0.58 | C1 |  |
| KLF9 | 0.58 | C28 |  |  | SEMA3E | 0.58 | C1 | -1.8 |
| 2810003C17RIK | 0.58 | C1C2 |  |  | PVRL3 | 0.58 | C1 | 1.6 |
| CLMN | 0.58 | C1 | -1.8 |  | TSPAN2 | 0.58 | C1 |  |
| SLC23A2 | 0.58 | C1C28 |  |  | LRP2 | 0.58 | C1C2C28 | -2.7 |
| SH3D19 | 0.58 | C1C28 |  |  | COL6A2 | 0.58 | C1 |  |
| UPK3B | 0.58 | C1 |  |  | PNLIPRP1 | 0.58 | C2 | -10.7 |
| PCOLCE2 | 0.58 | C1 |  |  | LPP | 0.58 | C1 |  |
| PALM2 | 0.58 | C1C2 | -1.9 |  | B3GNT2 | 0.58 | C1 |  |
| TSN | 0.58 | C1 |  |  | NLK | 0.58 | C1 |  |
| JAM2 | 0.58 | C1 |  |  | MID1IP1 | 0.58 | C2 |  |
| FBLIM1 | 0.58 | C1 |  |  | ZDHHC14 | 0.58 | C1 | -1.6 |
| SEMA3E | 0.58 | C1 | -1.8 |  | ABCD3 | 0.58 | C1 |  |
